# Molecular docking study of potential phytochemicals and their effects on the complex of SARS-CoV2 spike protein and human ACE2

**DOI:** 10.1038/s41598-020-74715-4

**Published:** 2020-10-19

**Authors:** Anamika Basu, Anasua Sarkar, Ujjwal Maulik

**Affiliations:** 1 Department of Biochemistry, Gurudas College, Calcutta, India; 2 Computer Science and Engineering Department, Jadavpur University, Calcutta, India

**Keywords:** Computational chemistry, Virtual drug screening

## Abstract

Angiotensin converting enzyme 2 (ACE2) (EC:3.4.17.23) is a transmembrane protein which is considered as a receptor for spike protein binding of novel coronavirus (SARS-CoV2). Since no specific medication is available to treat COVID-19, designing of new drug is important and essential. In this regard, in silico method plays an important role, as it is rapid and cost effective compared to the trial and error methods using experimental studies. Natural products are safe and easily available to treat coronavirus affected patients, in the present alarming situation. In this paper five phytochemicals, which belong to flavonoid and anthraquinone subclass, have been selected as small molecules in molecular docking study of spike protein of SARS-CoV2 with its human receptor ACE2 molecule. Their molecular binding sites on spike protein bound structure with its receptor have been analyzed. From this analysis, hesperidin, emodin and chrysin are selected as competent natural products from both Indian and Chinese medicinal plants, to treat COVID-19. Among them, the phytochemical hesperidin can bind with ACE2 protein and bound structure of ACE2 protein and spike protein of SARS-CoV2 noncompetitively. The binding sites of ACE2 protein for spike protein and hesperidin, are located in different parts of ACE2 protein. Ligand spike protein causes conformational change in three-dimensional structure of protein ACE2, which is confirmed by molecular docking and molecular dynamics studies. This compound modulates the binding energy of bound structure of ACE2 and spike protein. This result indicates that due to presence of hesperidin, the bound structure of ACE2 and spike protein fragment becomes unstable. As a result, this natural product can impart antiviral activity in SARS CoV2 infection. The antiviral activity of these five natural compounds are further experimentally validated with QSAR study.

## Introduction

COVID-19 is a deadly disease, where the infection is caused by SARS-CoV-2. The coronavirus particles are spherical in shape having spike proteins around them. These proteins are responsible for virus replication in human host cells. Spike proteins after attaching with human cells, undergo structural changes, which results in a fusion of viral particle membrane with human host cell membrane. Thus, the viral RNA enters into the host cell and produces more viruses after copying its genome. SARS-CoV-2 spike proteins bind to the receptor proteins, on the host cell surface, known as angiotensin converting enzyme 2 (ACE2). The molecular level structure of SARS-CoV-2 spike protein has a Receptor Binding Domain (RBD) for binding to host human cells. Receptor Binding Domain (RBD) of spike glycoprotein interacts with ACE2 receptor in Protease Domain (PD) of the host human cell, causing viral infection.

Considering the preliminary data, it has been suggested that ACE2 is a membrane protein for SARS-CoV-2, that is identified to cause the respiratory disease outbreak in Wuhan in late 2019^1,2^. Specifically, SARS-CoV-2 is a beta coronavirus, having similarity with SARS- CoV virus, in binding with human ACE2 receptor and spike glycoprotein for viral entry^3^. Tai et al.^3^ suggested that RBD fragment (from amino acid residues 331–524 of spike protein) in SARS-CoV-2 strongly binds with human ACE2 (hACE2) as well as bat ACE2 (bACE2) receptors. Thus, this spike protein fragment is responsible for the entry of both SARS-CoV-2 and SARS-CoV in human ACE2-expressing cells. Small molecules, which can affect the binding efficiency of spike protein with its receptor, may act as the viral attachment inhibitor for both infections.

Every coronavirus comprises four structural proteins namely spike, envelope, nucleocapsid and membrane proteins. Among them, spike (S) protein is the most vital protein which controls the biological processes such as viral particle attachment, fusion and lastly entry in the host cell. As a result, it can be considered as a target for development of medicines in COVID-19, as well as SARS-CoV infection^4,5^. The S protein facilitates the entry of virus in human host cells. Initially it binds to ACE2 protein through its receptor-binding domain. Subsequently, it fuses with the viral and host membranes. However SARS-CoV-2 spike protein is about 10–20 times more probable to bind with ACE2 on human cells, compared to that of spike protein from the SARS-CoV infection (occurred in 2002).

**Figure 1:**
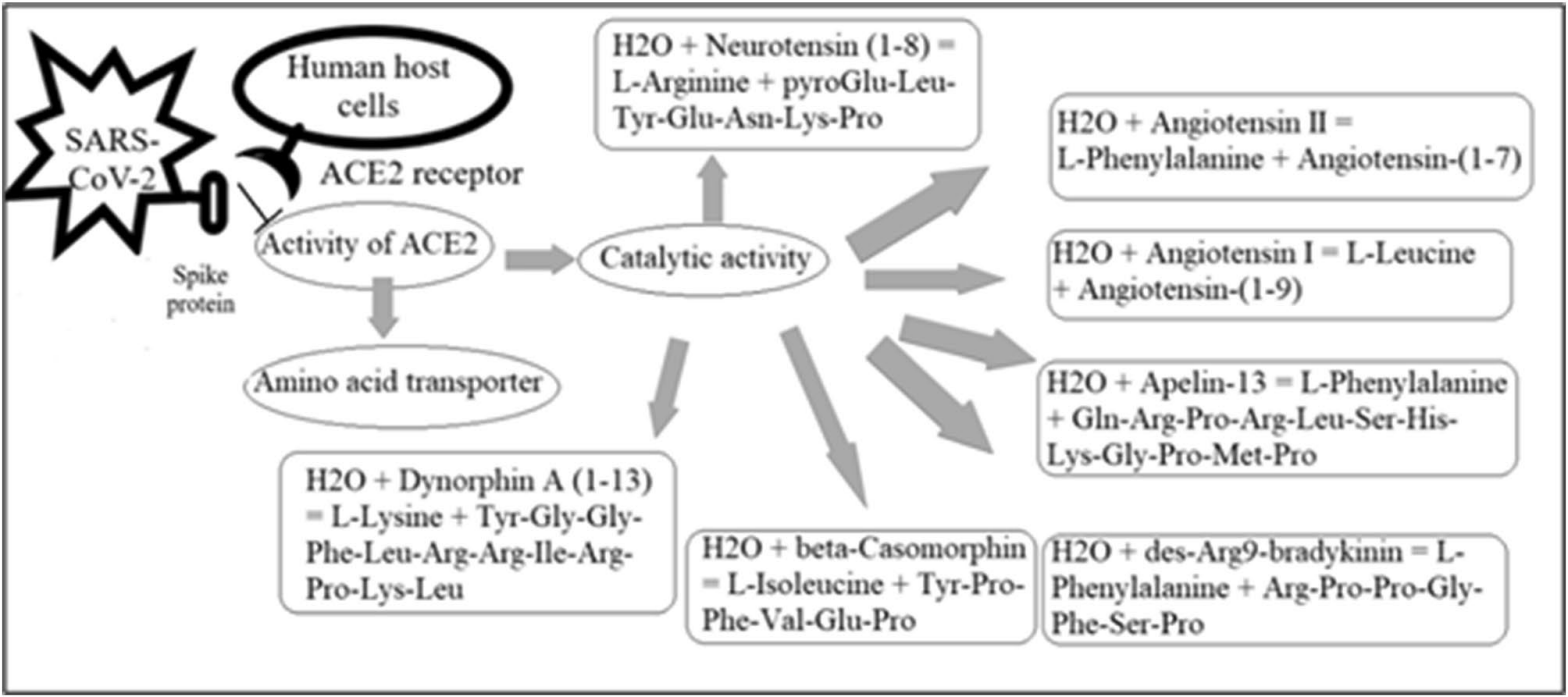
Different activities of ACE2 protein and inhibitory role of spike protein.

ACE2 is a membrane bound receptor for both coronaviruses such as SARS-CoV and SARS-CoV-2. For the first infection, ACE2 is confirmed as receptor from both ‘in vitro’ as well as ‘in vivo’ studies^6^. Similarly, Zhou et al.^7^ has experimentally demonstrated that ACE2 is cellular entry receptor for SARS-CoV-2 in human host. ACE2 enzyme having catalytic activity in maturation reaction of angiotensin, which is a peptide hormone. ACE2, a type I membrane bound protein, is expressed in many tissues including heart, kidney, intestine except lungs. ACE2-expressing epithelial cells express several viral replication associated genes^8^, signifying that these cells can facilitate coronavirus replication in the lung^9^. The presence of ACE2 receptor in other tissues, can explain the cause of kidney damage, heart failure and liver damage in COVID-19 infected patients. Different activities of ACE2 protein and inhibitory role of spike protein, are depicted in Fig. 1.

**Figure 2:**
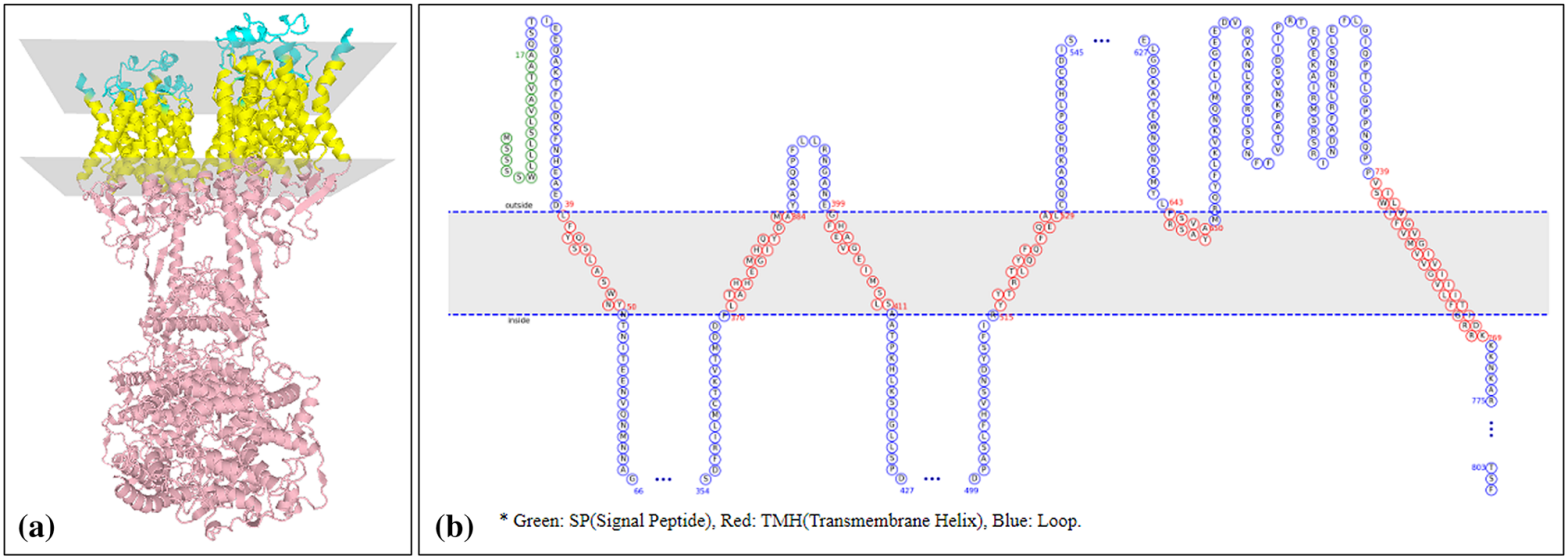
Topology of membrane protein ACE2 (**a**) from PDBTM (PDB ID 6M18) and (**b**) from MemBrain 3.1.

Schematic diagram for structure of transmembrane ACE2 protein is shown in Fig. 2. There are three topological domains in ACE2, such as extracellular signal sequence, a transmembrane helical domain and a cytoplasmic domain. Figure 2a presents the topology of membrane protein ACE2 as present in PDB ID 6M18 according to Protein Data Bank of Transmembrane Proteins database (PDBTM)^10^. In Fig. 2b, a representative diagram of ACE2 protein is shown, after analyzing with MemBrain 3.1, web server^11^.

Several potential therapeutic approaches have been experimented to treat SARS-CoV-2 infection such as protein-based vaccine design, blocking of ACE2 receptor and effect of phytochemicals on spike protein binding with its ACE2 receptor. Among the various therapeutic strategies that have been proposed for the treatment of SARS-Co V 2 treatment, drug designing with phytochemicals is a well-known method. Several phytochemicals for example, *Ocimum sanctum* extract on main protease protein^12^, 5,7,3′,4′-tetrahydroxy-2′-(3,3-dimethylallyl) isoflavone from *Psorothamnus arborescens* on 3-chymotrypsin-like protease^13^ and curcumin, brazilin, and galangin from *Curcuma *sp., *Citrus *sp., *Alpinia galanga*, and *Caesalpinia sappan* on both SARS-CoV-2 protease and RBD of spike protein^14^ and Belachinal, Macaflavanone E and Vibsanol B on envelop protein^15^ are analyzed with the help of molecular docking and molecular dynamics studies. In the last study, hesperidin, one of common flavonoids in Citrus sp., has been selected as potent inhibitor with the lowest binding score and the highest binding affinity for different protein receptors.

Wu et al.^16^ have used homology modeling technique to model several viral proteins and at the same time, two human target proteins. They have screened probable small ligand molecules from the ZINC Drug as well as traditional Chinese medicine and natural products databases to identify potential molecules to treat SARS-CoV-2 infection. Hesperidin molecule, which is known for its anti-oxidant effect and anti-inflammatory, is obtained from *Citrus aurantium*. In their study, it was observed Hesperidin can only bind to the interface between Spike protein and ACE2 receptor. So, they have suggested that Hesperidin can disrupt the interaction of ACE2 and receptor-binding domain. But, during molecular docking analysis, they used PDB file SARS_CoV-2 _Spike_RBD_homo_Hesperidin considering RBD-S (PDB ID: 6LXT) and PD-ACE2 (PDB ID: 6M18). PDB structure of 6LXT contains structure of post fusion core of 2019-nCoV S2 subunit in dimeric form along with tetraethylene glycol and Zn^2+^ ion. Similarly, PDB ID: 6M18 represents angiotensin-converting enzyme 2 in complex with sodium-dependent neutral amino acid transporter B (0) AT1. There are several experimental structures which have been discovered, where spike protein is present as a whole with its receptor ACE2 in RSCB database. But, all of them have some small molecules present as ligands or some other macromolecules e.g. PDB ID 6CS2 containing fibritin. Similarly, PDB ID 6M17 shows the presence of sodium-dependent neutral amino acid transporter B(O)AT1 in its structure. In this study, specifically the 3D structure of spike protein (from amino acid sequence 331–524) and its binding site with its host cell receptor ACE2 protein, are focused. So, homology modeling structure of spike protein is required for molecular docking study.

Since, both SARS-CoV-2 spike protein and SARS-CoV spike protein can bind with human host ACE2 receptor protein, literatures are searched for binding inhibitor for EC 3.4.17.23—angiotensin-converting enzyme 2 (ACE2) in PubMed^17^. Ho et al.^18^ showed that, 1,3,8-trihydroxy-6-methylanthraquinone (emodin) can block interaction between SARS-CoV spike protein and ACE2, with 94.12% inhibition at 0.05 mM. 1,8 dihydroxy-3-carboxyl-9,10-anthraquinone or rhein and anthraquinone exhibit slight inhibition in spike protein binding. But, 5,7-dihydroxyflavone or chrysin can act as a weak inhibitor.

**Figure 3:**
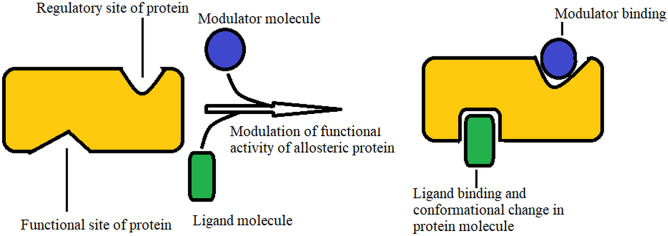
Functions of allosteric protein.

A protein may contain two binding sites for two different ligands. Under this circumstance, noncovalent ligand binding in the first site may alter the shape and thus the binding characteristics of second binding site. This biological phenomenon is known as allosteric modulation (as shown in Fig. 3). This protein with two binding sites is termed as allosteric protein. The first and second binding sites of that allosteric protein, are the functional (or active) and regulatory sites respectively. The first site carries out the protein’s physiological function (shown in Fig. 3). The ligand in second binding site is the modulator molecule, which allosterically modulates the shape and thus regulating the functional site activity of that protein.

**Figure 4:**
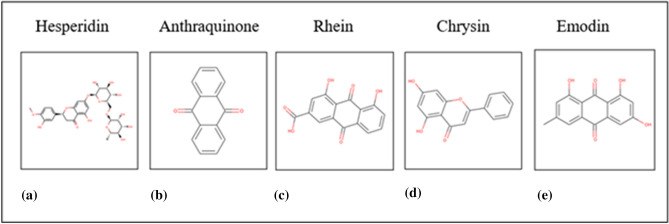
Structures of phytochemicals.

When spike protein fragment as ligand binds with ACE2 protein, this ACE2 protein functions as virus receptor, participating in the biological process known as the viral particle entry in host cell. Due to presence of a modulator molecule, for example hesperidin, ligand binding activity of spike protein, allosterically may be modulated. Some phytochemicals, which have been reported earlier as spike protein inhibitor for SARS^17,18^ are considered here as small molecules for protein–ligand molecular docking study. These phytochemicals are present in Indian medicinal plants. Name, source, chemical class and structures of phytochemicals e.g. hesperidin, emodin, anthraquinone, rhein and chrysin are enlisted in Table 1 and Fig. 4. This information is collected from the curated database—IMPPAT: Indian Medicinal Plants, Phytochemistry and Therapeutics^19^. To study the effect of Indian phytochemicals on spike protein fragment, molecular docking study is used for spike glycoprotein fragment with human ACE2 receptor. Bound structure of spike glycoprotein with human ACE2 receptor is considered here as target molecule for treatment of COVID-19 and the phytochemicals are considered here as modulators. These chemical compounds can bind with the host protein ACE2 as non-competitive molecule and impart their anti-viral activity by destabilizing spike protein binding with human host ACE2 receptor.

**Table 1:** Phytochemicals and their Indian medicinal plant sources.

Indian medicinal plant	Phytochemical identifier	Phytochemical name	Chemical class of phytochemicals
*Valeriana Jatamansi*	CID:10621	Hesperidin	Flavonoid glycoside
*Cassia Angustifolia*	CID:6780	Anthraquinone	Anthraquinone
*Oroxylum Indicum*	CID:6780	Anthraquinone	Anthraquinone
*Cassia Angustifolia*	CID:10168	Rhein	Anthraquinone derivative
*Oroxylum Indicum*	CID:5281607	Chrysin	Flavone
*Rheum Emodi*	CID:3220	Emodin	Anthraquinone derivative

## Results

### Protein molecular modeling of spike protein fragment

Gene Bank accession number for SARS-CoV-2 S is QHR63250.2, LOCUS QHR63250, Accession MN996527.1is used for protein molecular modeling of spike protein fragment.

Primary amino acid sequence of spike protein fragment (331–524) is as follows.

NATRFASVYAWNRKRISNCVADYSVLYNSASFSTFKCYGVSPTKLNDLCFTNVYADSFVIRGDEVRQIAPGQTGKIADYNYKLPDDFTGCVIAWNSNNLDSKVGGNYNYLYRLFRKSNLKPFERDISTEIYQAGSTPCNGVEGFNCYFPLQSYGFQPTNGVGYQPYRVVVLSFELLHAPATV.

### Primary and secondary structure analyses of spike protein fragment

The analysis of primary structure of the spike protein fragment SARS-CoV-2 shows that it has 193 amino acid residues. Similarly, secondary structure analysis with PDBsum^20^, shows that this protein fragment contains 3 sheets, 1 beta hairpin, 2 beta bulges, 9 strands, 6 helices, 1 helix-helix interaction, 14 beta turns, 4 gamma turns and 2 disulfide bonds.

### 3D structure modeling and validation

3D structure of S protein fragment has been modeled by using SWISSMODEL^21^ server. Template 6lzg.1. B is selected for modeling protein with the sequence identity 100% and coverage 100% compared to the other two templates (Table 2) for modeling.

**Table 2:** Templates for 3D structure of the spike protein fragment.

Template	Seq identity	Oligo-state	Found by	Method	Resolution	Seq similarity	Coverage	Description
6lzg.1. B	100.00	Monomer	HHblits	X-ray	2.50 Å	0.62	1.00	SARS-CoV-2 Spike receptor-binding domain
6m0j.1.B	100.00	Monomer	HHblits	X-ray	2.45 Å	0.62	1.00	SARS-CoV-2 receptor-binding domain
6w41.1.C	100.00	Monomer	HHblits	X-ray	3.08 Å	0.62	1.00	Spike glycoprotein receptor binding domain
6m17.1.C	100.00	Monomer	HHblits	EM	NA	0.62	1.00	SARS-coV-2 Receptor Binding Domain

The SWISS-MODEL template library (SMTL version 2020-04-08, PDB release 2020-04-03) is searched with BLAST^22^ and HHBlits^23^ for phylogenetically matched structures with the target sequence in Table 2. Overall, 101 templates are found.

**Figure 5:**
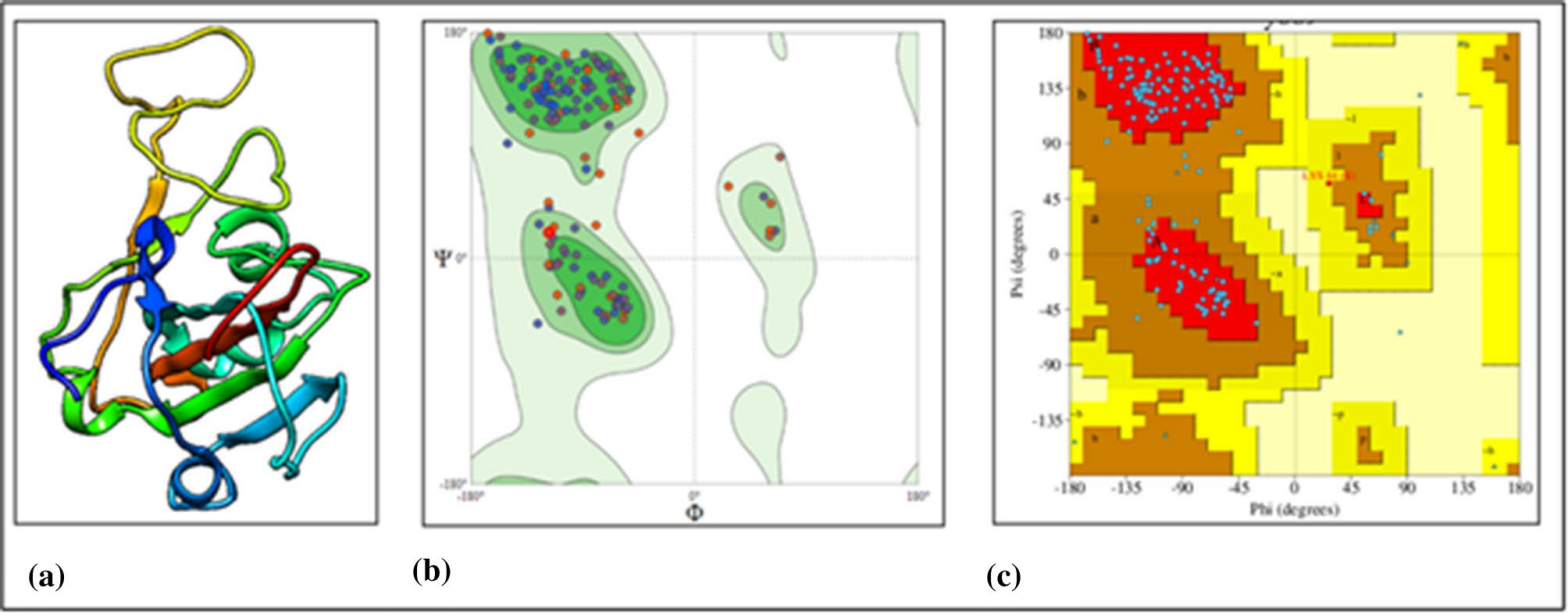
(**a**) 3D structure for spike protein fragment Rmachandran plot, (**b**) from MolProbity server, (**c**) PROCHECK server.

Modelled structure obtained from SWISSMODEL server^21^ has − 2.87 QMEAN score, shown in Fig. 5a. QMEAN value is a linear combination of four statistical relevant terms. It provides a Z score to relate it to high resolution X-ray structures with similar size. Higher Z score is related to more favorable model. Ramachandran plots are drawn for this model by using two web servers e.g. Molprobity^24^ and PDBsum^20^, as shown in Fig. 5b,c. For this model the overall average value of G-factors is − 0.18, which is not unusual for main-chain covalent forces and dihedral angles. The value of G-factors shows a measurement for unusualness or out-of-the ordinary property. From MolProbity version 4.4^25^ it is calculated that, modelled structure has 94.44% residues in favored regions, 0.56% residues in outlier region and 3.18% in rotamer outlier region. Ramachandran plot statistics from PDBsum^20^ for modelled structure of spike protein fragment shows that, the residues in most favored regions [A, B, L], additional allowed regions [a, b, l, p], generously allowed regions [~ a, ~ b, ~ l, ~ p] and disallowed regions [X, X] are 136 (86.1%), 21 (13.3%), 1 (0.6%) and 0 (0.0%) respectively.

### Molecular docking between spike protein fragment and human ACE2 receptor

Human ACE2 receptor (PDB ID 1R42)^26^ is considered as receptor protein for molecular docking study of spike protein fragment with its receptor in human host.

**Figure 6:**
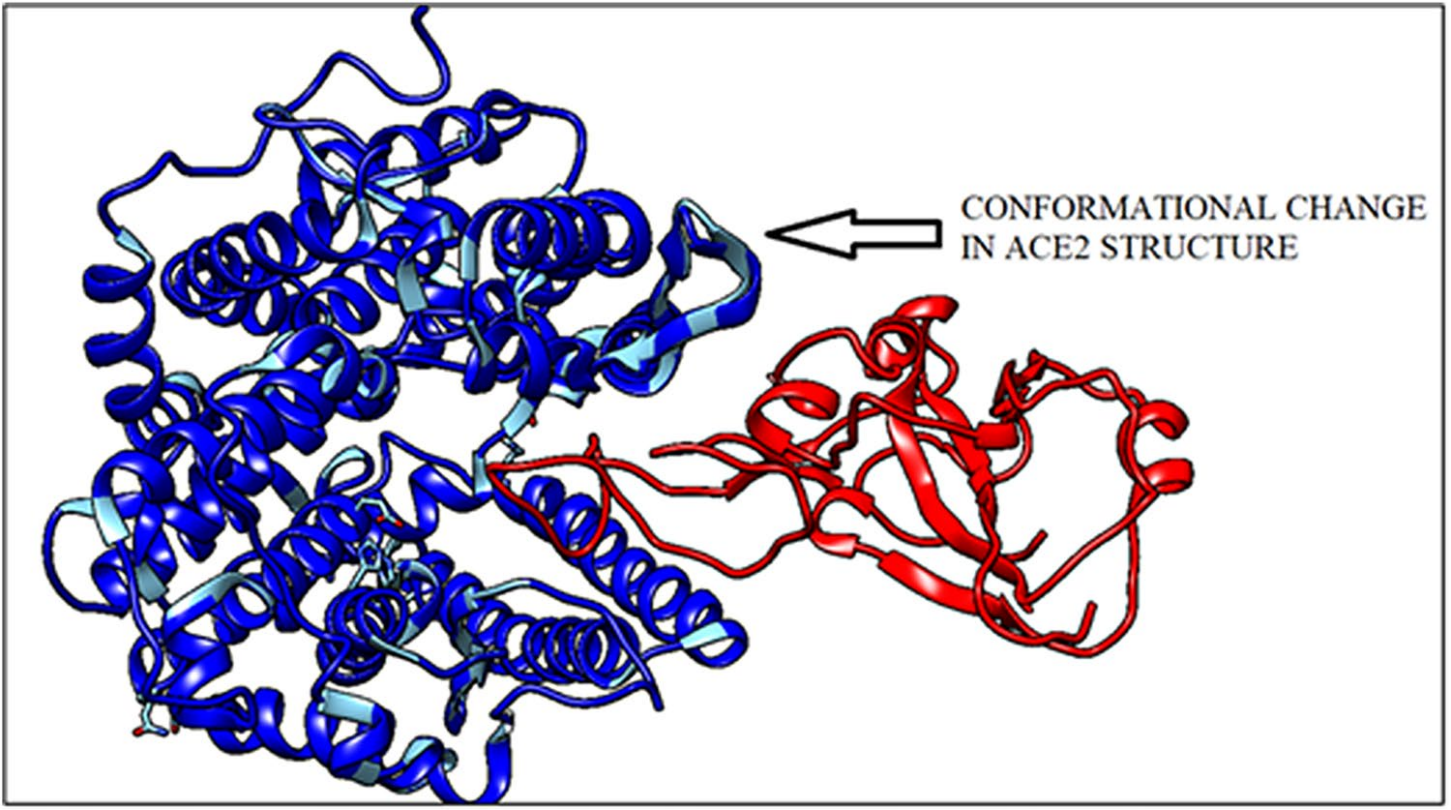
SARS CoV2 S protein binding with human ACE2 receptor protein (generated by using UCSF Chimera software^45^).

By using ClusPro^27^ web server, docking structure of A chain of human ACE2 receptor binds with SARS CoV2 spike protein fragment with binding energy − 779.8 kcal/mole. When ACE2 receptor protein binds with S protein fragment, a conformational change occurs (shown in Fig. 6).

**Figure 7:**
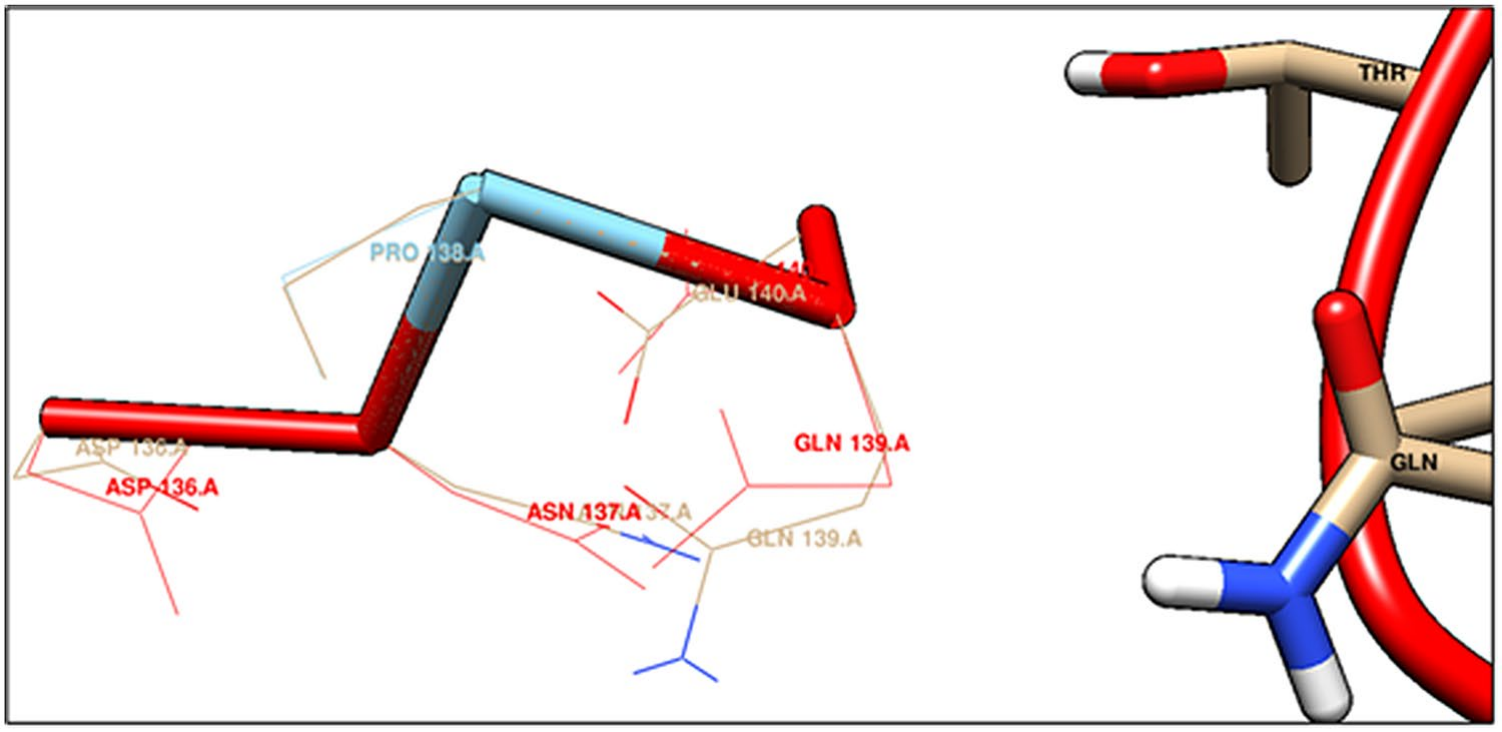
Distorted amino acids after spike protein binding in ACE2 receptor (generated by using UCSF Chimera software^45^).

Amino acids present in distorted site of ACE2 are ASP136, ASN 137, PRO 138, GLN139 and interacting amino acids of spike protein fragment are GLN 403, LYS 451 and ASP 416 (Fig. 7).

From molecular docking study, a 3D structural figure with more detailed focus on ACE2-S protein interface, is presented in Supplementary File S1 (Supplementary Figures S1.1, S1.2). Detailed analysis of intermolecular contacts (ICs) and list of amino acids which are present in the protein–protein interaction sites of spike protein fragment with ACE2 receptor have been carried out and are accessible in Supplementary File S1 (Supplementary Tables S1.2, Table S1.3).

Bound structure of SARS CoV2 spike protein fragment with ACE2 receptor protein is considered as therapeutic target for SARS-CoV2 treatment.

### Molecular docking study of phytochemicals from Indian medicinal plants

#### Spike protein binding with ACE2 in presence of hesperidin

**Figure 8:**
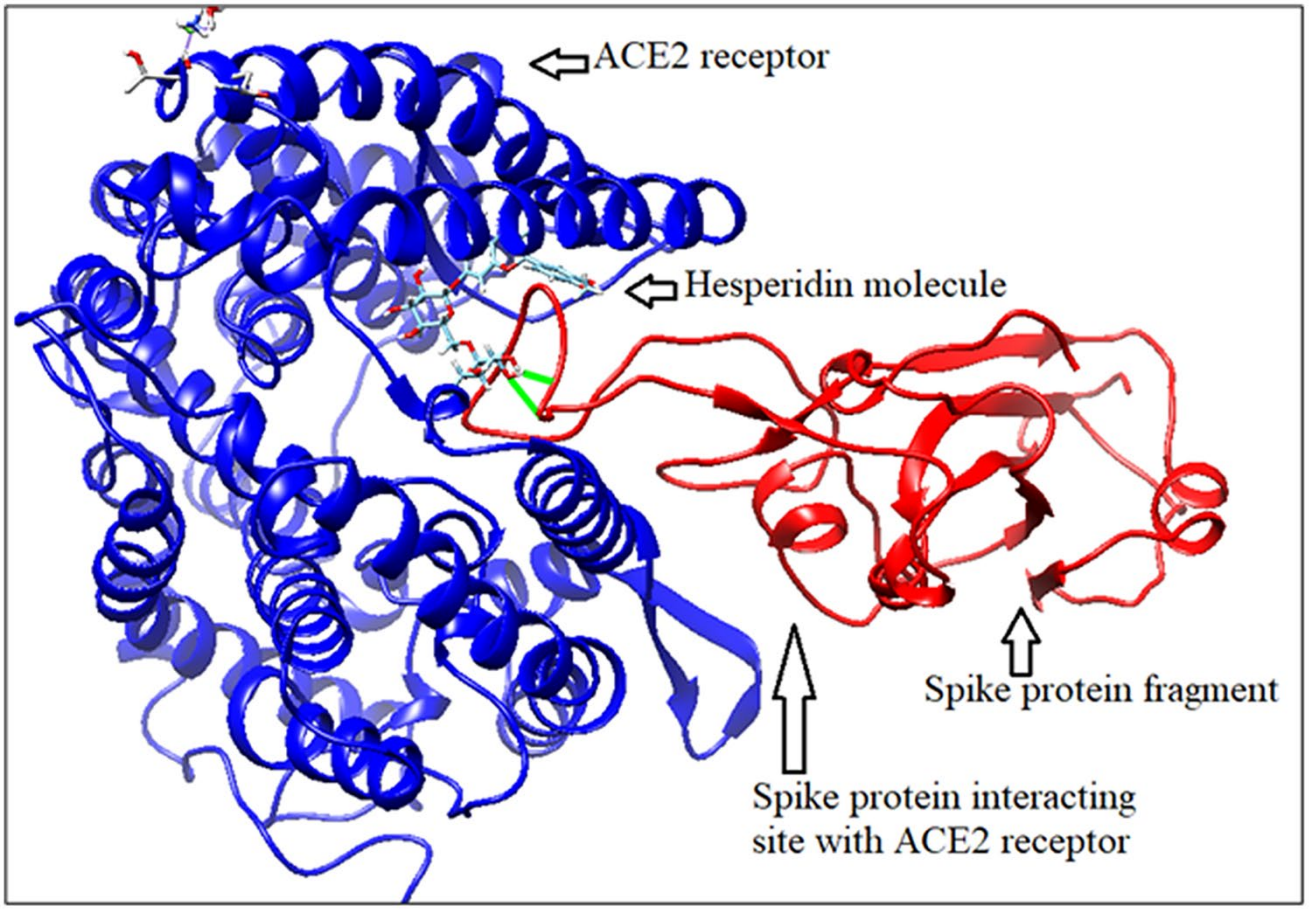
Spike protein binding with ACE2 in presence of hesperidin (generated by using UCSF Chimera software^45^).

In Fig. 8, spike protein fragment (331–524) is shown in red colour, hesperidin molecule in stick model and human ACE2 is shown in blue colour. Hesperidin binds with spike protein fragment and its receptor ACE2 with binding energy − 8.99 kcal/mole. This docked structure is stabilized by two H binding (shown in Figure with green lines) at PHE 457 of spike protein with O7 atom of hesperidin, with bond length 2.618 Å and H atom of small molecule hesperidin with O atom of GLU 455 of spike protein fragment with a distance 2.067 Å. Hesperidin binds at ASN 63, ALA 71, LYS 74 and SER 44 amino acids of ACE2. Detailed analysis of intermolecular contacts (ICs) and list of amino acids which are present in the protein–protein interaction sites of spike protein fragment with ACE2 receptor in absence and presence of hesperidin have been carried out and are presented in Supplementary File 1 (Figure S1.1, S1.2; Tables S1.2, Table S1.3).

#### Spike protein binding with ACE2 in presence of emodin

**Figure 9:**
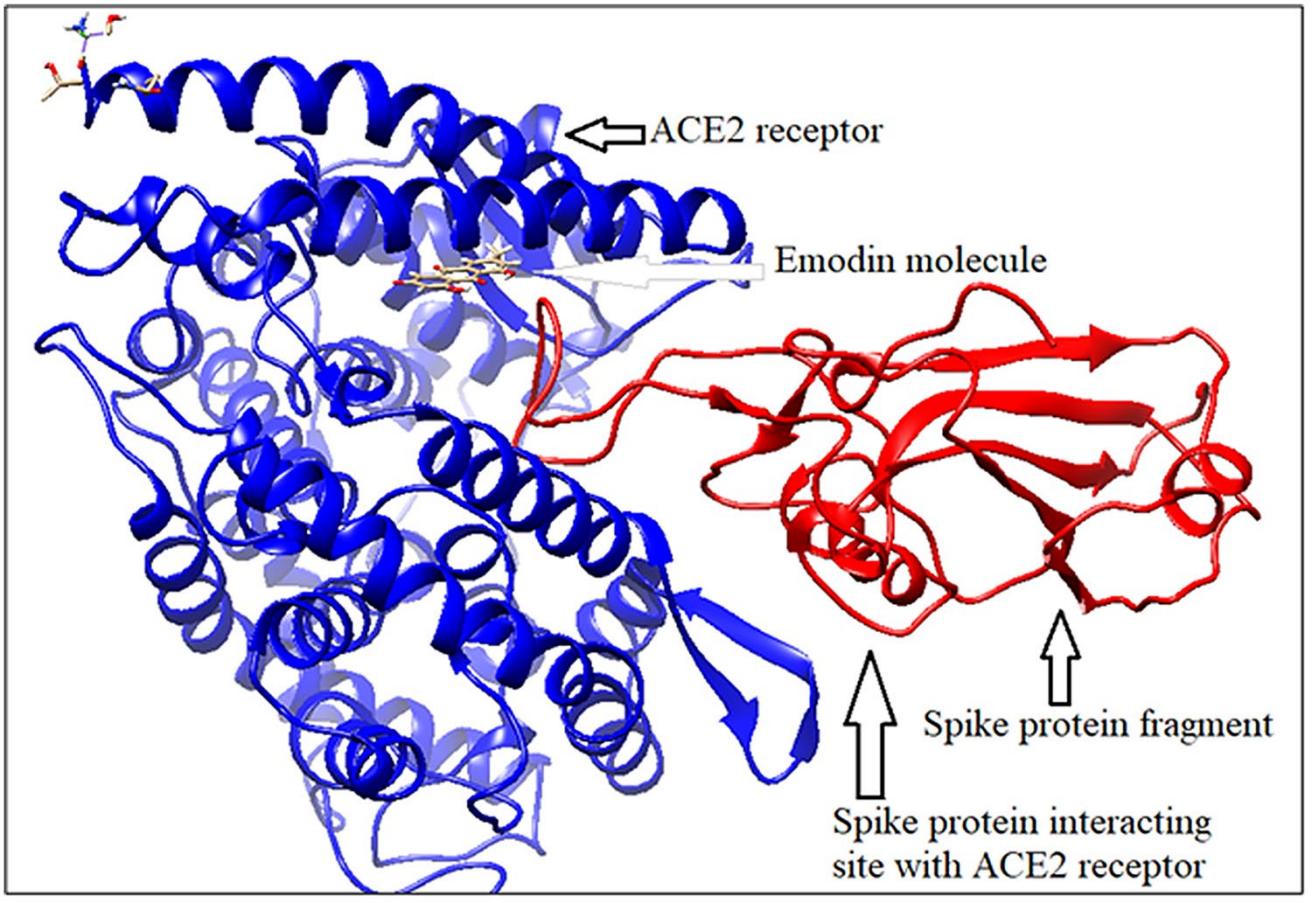
Spike protein binding with ACE2 in presence of emodin (generated by using UCSF Chimera software^45^).

The phytochemical emodin, obtained from *Rheum emodi* or Himalayan rhubarb^28^, binds with spike protein fragment and its receptor human ACE2 protein^29^, at the same cleft (Fig. 9), similar to that of hesperidin. But binding energy is less for emodin binding (− 6.19 kcal/mole) compared to that of hesperidin (− 8.99 kcal/mole).

#### Spike protein binding with ACE2 in presence of anthraquinone

**Figure 10:**
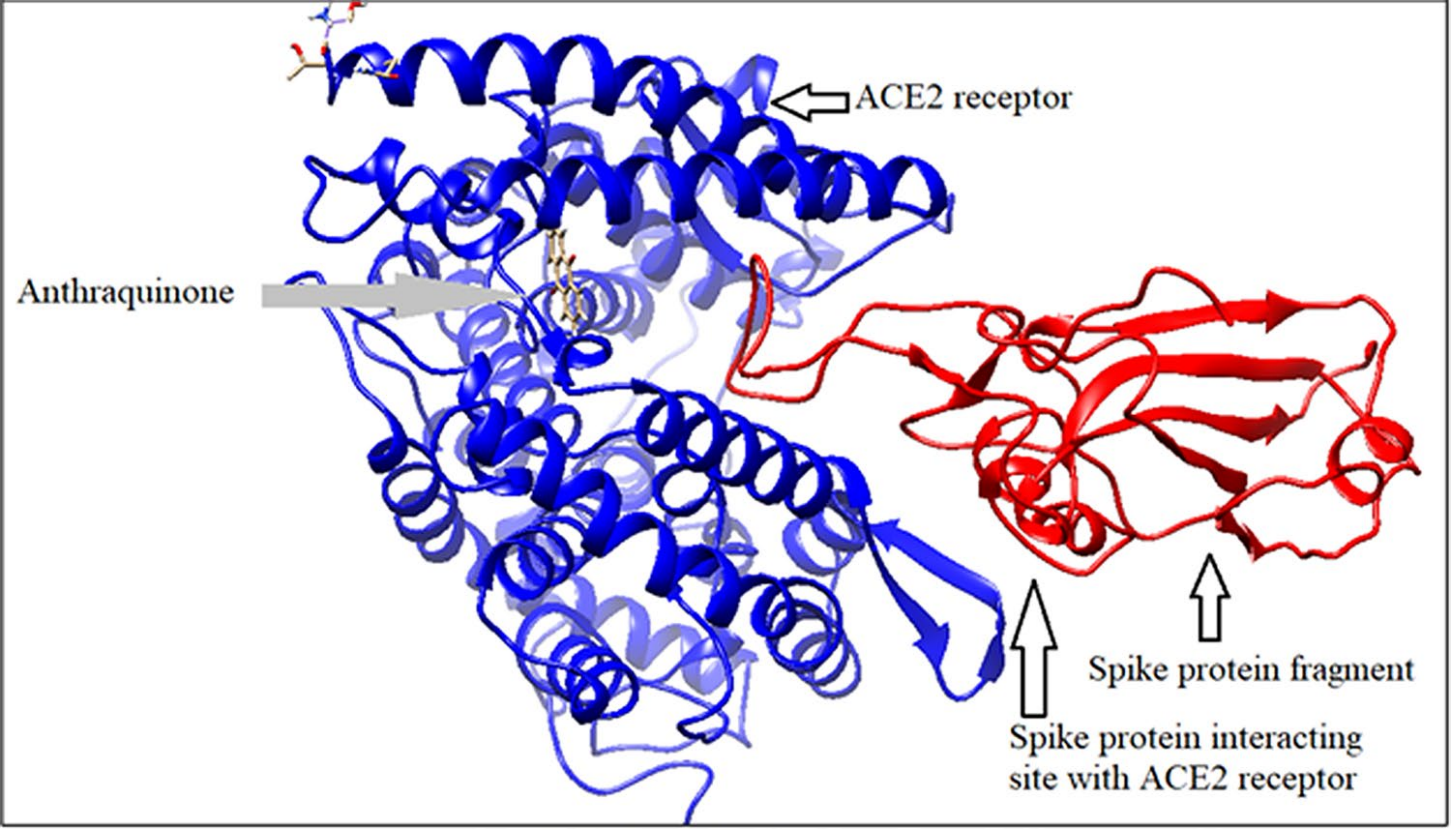
Spike protein binding with ACE2 in presence of anthraquinone (generated by using UCSF Chimera software^45^).

Though anthraquinone can bind with bound structure of spike protein fragment and its receptor ACE2 molecule, by releasing binding energy − 6.15 kcal/mole, the binding site of this phytochemical is totally different from that of hesperidin and emodin (Fig. 10).

#### Rhein binding with bound spike protein and ACE2 receptor protein

**Figure 11:**
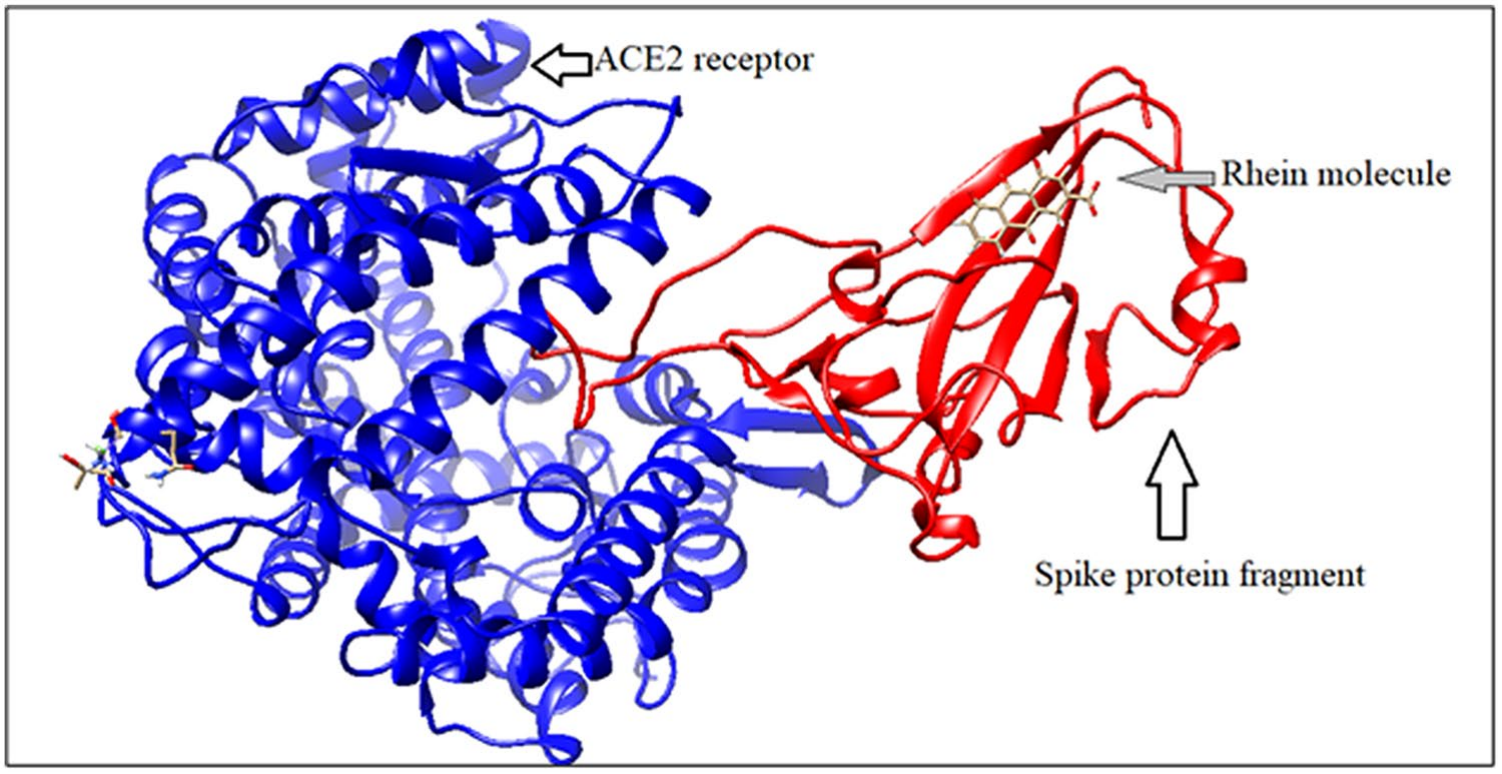
Rhein binding with bound spike protein fragment and ACE2 (generated by using UCSF Chimera software^45^).

The phytochemical rhein binds with docked structure of spike fragmented protein and human ACE2 receptor with ΔG value − 8.73 kcal/mole. But, the binding site of this chemical is totally different from earlier substances (Fig. 11). Rhein can bind with only spike protein fragment. It has no interaction with human ACE2 receptor protein molecule.

#### Chrysin binding with bound spike protein fragment and ACE2 receptor

**Figure 12:**
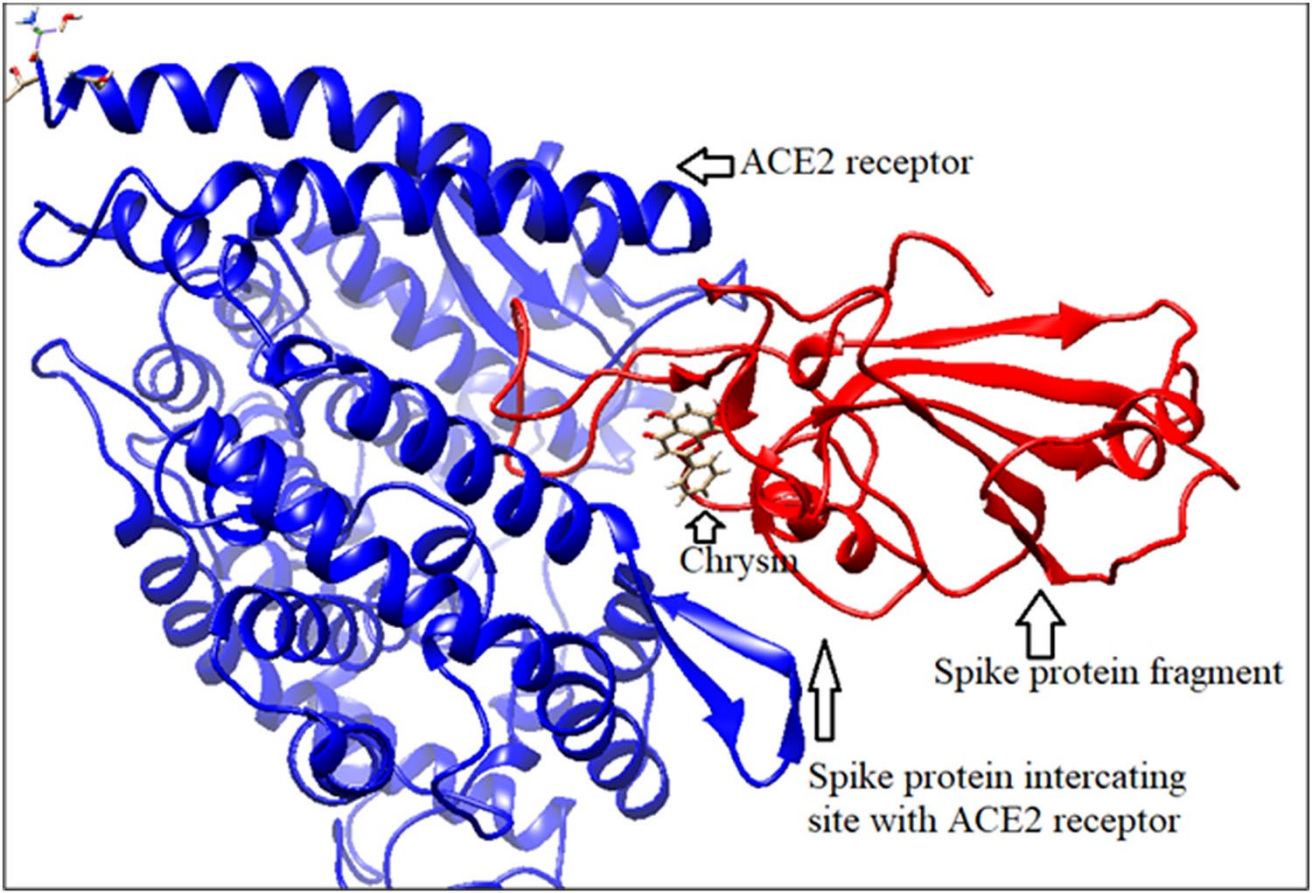
Chrysin binding with bound spike protein fragment and ACE2 (generated by using UCSF Chimera software^45^).

**Figure 13:**
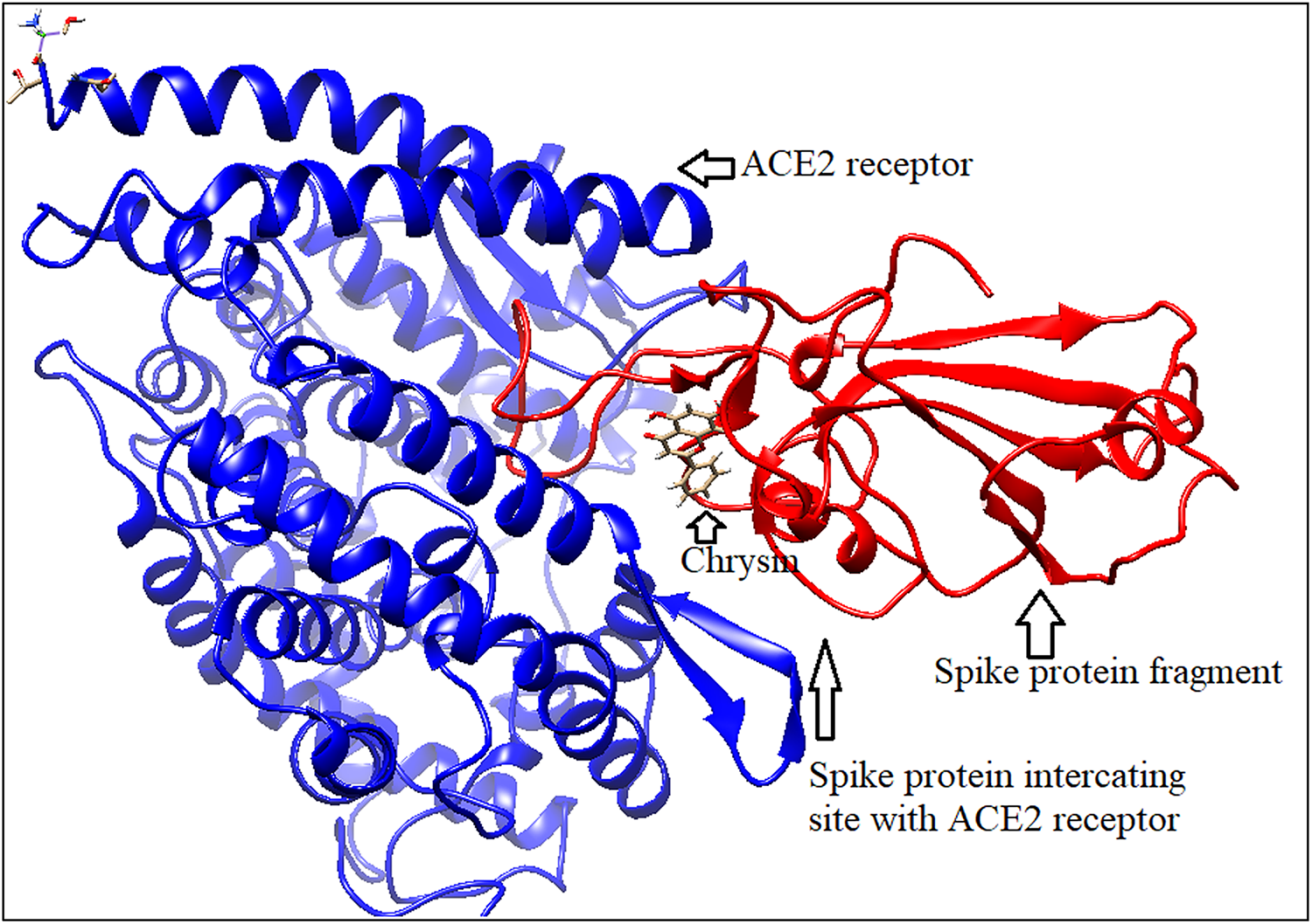
Chrysin binding cleft (generated by using UCSF Chimera software^45^).

Chrysin binds with the spike protein fragment and its ACE2 receptor with binding energy − 6.87 kcal/mole (Fig. 12). This phytochemical binding site is almost similar with that of spike protein fragment molecule and its receptor. A conformational change occurs in ACE2 receptor molecule after spike protein fragment binding. Chrysin binding cleft is located near to that site as shown in Fig. 13.

#### Comparative study of binding of ACE2 with SARS-CoV-2 S protein in presence of known inhibitors of coronavirus spike proteins and five natural compounds

**Figure 14:**
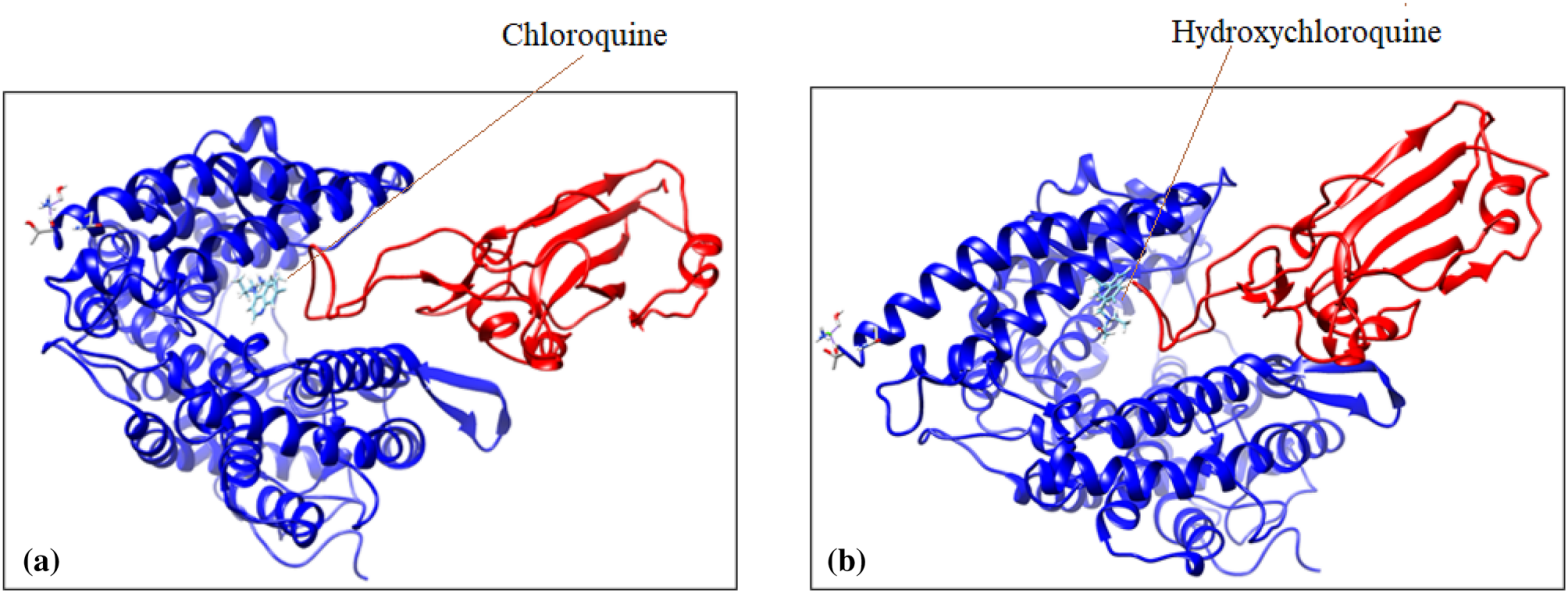
Docking structure of ACE2 bound with spike protein fragment in presence of (**a**) chloroquine (**b**) hydroxychloroquine (generated by using UCSF Chimera software^45^).

Binding energy of chloroquine and hydroxychloroquin with the bound structure of ACE2 and spike protein fragment are − 8.99 kcal/mole and − 7.83 kcal/mole, respectively. Among these two well inhibitors of spike protein, hydroxychloroquine is bound with docking structure of ACE2 and spike protein fragment almost exactly at the same position, compared to that of hesperidin (shown in Fig. 14). Their binding energies are also comparable to that of hesperidin (Table 3).

**Table 3:** Energy parameters of bound structure of phytochemicals.

Name of phytochemicals	Energy/ Simple fitness	FullFitness	ΔG_vdw_	ΔG (Kcal/mole)
Hesperidin	59.4535	− 2147.5469	− 52.5659	− 8.99
Emodin	19.599	− 2301.9927	− 23.3637	− 6.19
Anthraquinone	17.7976	− 2234.7346	− 21.5368	− 6.15
Rhein	36.5174	− 2310.458	− 107.401	− 8.73
Chrysin	15.8545	− 2266.9272	− 31.1973	− 6.87
Chloroquine	-3.75883	− 2295.2732	− 74.6772	− 8.98
Hydroxychloroquine	4.67143	− 2272.3052	− 40.848	− 7.82

Energy parameters of bound structure of phytochemicals with spike protein fragment and ACE2 receptor are shown in Table 3.

Considering the lowest binding energy, the phytochemical hesperidin is considered as the most suitable ligand for target molecule, which is formed by binding of spike protein fragment with its human host ACE2 receptor. Similarly, the protein–protein interaction energy of spike protein fragment bound with ACE2 receptor, has been marginally changed (15.4–14.5 kcal/mole) after their binding with phytochemical hesperidin as shown in Supplementary File S1 (Supplementary Table S1.1).

In six docking structures interacting amino acids of ACE2 receptor and spike protein fragment are summarized in Table 4.

**Table 4:** Interacting amino acids in docking structures.

Docking structure	Interacting amino acids of ACE2 receptor	Interacting amino acids of spike protein fragment
Spike protein fragment with ACE2	ASP136, ASN 137, PRO 138, GLN 139	GLN 403, LYS 451, ASP 416
Hesperidin binding with spike protein and ACE2	ASN 63, ALA71, LYS 74, SER 44	VAL 472, GLY 474, GLY 471, PHE 475, GLU 473
Emodin binding with spike protein and ACE2	ALA 71, ASP 67, LYS 74	VAL 472, GLY 474, ALA 464, ASN 448
Anthraquinone binding with spike protein and ACE2	SER 105, ASN 103, GLN 102, LEU 100, PHE 28	No interacting amino acids
Rhein binding with spike protein and ACE2	No interacting amino acids	SER 388, VAL 401, THR 333, ASN 332, ASN 353
Chrysin binding with spike protein and ACE2	THR 129, ILE 126, THR 125	ARG 443, SER 448, ASN 449, TYR 410, PHE 486, TYR 484, THR 487, ASN 488, LYS 406
Chloroquine binding with spike protein and ACE2	GLY 405, HIS 401, THR 347	THR 467, PRO 468, CYS 469
Hydroxychloroquine binding with spike protein and ACE2	ASP 67, ALA 71, SER 43	GLY 471, VAL 472, CYS 469

Considering the docking structures and interacting amino acids of both ACE2 receptor and spike protein fragment, chrysin can act as most competent inhibitor for spike protein binding with ACE2 receptor.

### Binding affinity calculation in different docking structures

The strength of protein–ligand binding in the binding site can be expressed as its binding affinity. The strength of attractive force of protein and ligand determines corresponding binding affinity. These binding affinity values of different docking structures of ACE2 and natural products, in absence and presence of spike protein fragment are calculated using Dockthor web server and enlisted in Table 5. In this soft docking method, standard algorithm precision is set with 1,000,000 evaluations, population size 750 and initial seed value of − 1985 for 24 runs.

**Table 5:** Binding affinity of ACE2 protein and different natural compounds as ligands.

Compound no.	Name of natural compounds	Affinity (Kcal/mole)	Total energy	VdW energy	Electrostatic energy
1	Hesperidin	− 9.167	55.969	− 29.393	− 22.905
2	Chrysin	− 7.146	9.875	− 9.783	− 25.985
3	Emodin	− 9.834	1.643	− 8.475	− 19.919
4	Anthraquinone	− 7.477	22.709	− 15.811	− 4.498
5	Rhein	− 7.423	10.453	− 10.247	− 28.256

The binding affinity values for five docking structures for ACE2 protein and five natural phytochemicals show that when spike protein of SARS CoV2 is not bound with its receptor, at that condition, all five compounds can form stable complex with ACE2 protein molecule.

Binding affinity of ACE2 protein and spike protein fragment as ligand and different natural compounds as modulators, are displayed in Table 6. Above results show that hesperidin molecule binds with ACE2 protein molecule (with binding affinity − 9.167 kcal/mole. But in case of the bound structure of ACE2 and spike protein fragment, hesperidin binding affinity changes to − 8.639 kcal/mole. This result indicates that due to presence of hesperidin, the bound structure of ACE2 and spike protein fragment becomes unstable. For emodin the trend in change of binding affinity is same (Table 6). As a result, this natural product can impart antiviral activity in SARS CoV2 infection as noncompetitive modulator. Anthraquinone and rhein are not considered as modulators from molecular docking study. But chrysin does not show the same antiviral activity.

**Table 6:** Binding affinity of ACE2 protein and spike protein fragment as ligand and different natural compounds as modulators.

Compound no.	Name of docking compound	Affinity	Total energy	VdW energy	Electrostatic energy
1	S_ACE2_hesperidin	− 8.639	63.528	− 27.816	− 12.045
2	S_ACE2_chrysin	− 8.009	10.130	− 19.542	− 12.273
3	S_ACE2_emodin	− 7.186	5.956	− 12.391	− 11.282
4	S_ACE2_anthraquinone	− 7.322	21.402	− 11.574	− 10.041
5	S_ACE2_rhein	− 6.954	17.775	− 12.177	− 17.482

Detailed analysis of Molecular dynamics simulation study of bound structure of ACE2 and spike protein fragment has been carried out and added in Supplementary Information S2. From molecular simulation study for various parameters, it can be concluded that hesperidin can act as modulator for the bound structure of ACE2 and spike protein fragment. This modulator molecule decreases the stability of the bound structure of ACE2 and spike protein fragment. This analysis confirms the antiviral activity of natural compound hesperidin as non-competitive allosteric modulator of spike protein binding with its human host receptor.

The experimental validation of the anti-viral activity of five natural compounds and their ability to inhibit the S protein-ACE2 interaction is verified by QSAR study. Detailed results for this QSAR study are exhibited in Supplementary Information S2.

## Discussion

Considering the primary sequence from 331 to 524 of Spike protein, a homology modelled structure is built using SWISSMODEL, with template 6lzg.1. B having sequence identity 100.00% and coverage 100%. This modelled structure is validated by Ramachandran plot. This stable spike protein fragment is used for binding with human host ACE2 receptor protein by molecular docking study.

Binding site of spike protein fragment with its ACE2 receptor lying on binding surface with interacting amino acids ASP 136, ASN 137, PRO 138 and GLN 139, forms a beta hairpin motif in between two β strands secondary structure (results from PDBsum). This binding site is present in extracellular domain of ACE2 protein.

Bound structure of SARS CoV2 spike protein fragment with ACE2 receptor protein is considered as therapeutic target for SARS-CoV2 treatment and screened with Indian phytochemicals e.g. hesperidin, emodin, anthraquinone, rhein and chrysin by molecular docking study. Among them, hesperidin binds with ASN 63, ALA71, LYS 74 of H2 helix and SER 44 of H1 helix of human ACE2 receptor protein noncompetitively in presence of spike protein fragment of SARS CoV2. Similarly, emodin binding amino acids i.e. ALA 71, ASP67 and LYS 74 are present on H2 helix of ACE2 molecule. Phytochemical anthraquinone interact with spike protein fragment and rhein has no interacting amino acids with ACE2 receptor. So, both of them are not considered as therapeutic agents in COVID treatment. But when chrysin binds with target molecule the interacting amino acids (THR 129, ILE 126 and THR 125), are located on H5 helix of ACE2 receptor protein.

The above mentioned β hair pin motif is a supersecondary structure. It comprises an antiparallel β sheet. Sequential segments of polypeptide chain form this β sheet, which are connected by a tight reverse turn. Here in ACE2 protein, this antiparallel β sheet is flanked by, in both sides with H5 and H6 helices of that protein. Globular protein ACE2 largely consists of straight runs in secondary structure. Stretches of a polypeptide joins this secondary structure, which change the direction abruptly. This β hair pin motif structures are present at the surface of the protein. Here the specific β hair pin motif contents amino acid sequences such as ASN134, Pro 135, ASP136 and ASN 137, which is present in the ligand binding site of ACE2 receptor protein. Proline is present as second residue, so that it can easily achieve the required conformation. This conformation has been changed due to binding of spike protein fragment. Distorted structure of ACE2 exposing ASP136, ASN 137, PRO 138, GLN 139 amino acids, can interact with GLN 403, LYS 451, ASP 416 of S protein of SARS-CoV2, at the ligand binding site of that protein.

FASTA alignment for PDB entry of spike protein fragment with 26 PDB entries, having at least a 30% sequence identity or E values < 0.001, has been executed in PDBsum^20^ (results are not shown here). Among three interacting amino acids of spike protein fragments GLN 403 and ASP 416 are well conserved among all sequences. But LYS 451 is conserved among SARS-CoV2 spike proteins and differed with ARG in SARS-CoV spike proteins. Though arginine is a positively charged, polar amino acid, it can be substituted with the other positively charged amino acid lysine. But a change from arginine to lysine is not always neutral. Arginine contains a complex guanidium group on its positively charged sidechain and shows a geometry and charge distribution, which is suitable for ideal binding with negatively charged amino acid residues. It can also form multiple hydrogen bonds. But lysine can also interact with negatively charged amino acid residues with a limited number of hydrogen bonds^30^.

In case of hesperidin, interacting amino acids of spike protein fragment e.g. VAL 472, GLY 474, GLY 471, PHE 475, GLU 473 are well conserved among PDB structures of SARS CoV-2 spike proteins (6m0j: E, 6lzg: B, 6w41:C, 6m17: E and 6vw1: E). But these residues are not present in structures of SARS-CoV spike glycoprotein structures (2dd8:S, 2ghw:A, 1q4z:A, 1t7g:A, 1xjp:A, 5xlr:A, 5x58:A, 6nb6:A, 6nb7:A, 6acc:A, 6acd:A , 6acg:A , 6acj:A, 6ack:A , 2ghv:E, 6waq:D, 5wrg:A, 3bgf:S, 5x5b:A, 6crw:A, 6crx:B, 6crz:A and 6cs0:A).

For emodin phytochemical, the interacting amino acids of spike protein fragment i.e. ALA 464 and ASN 448 are also conserved in five SARS CoV-2 spike protein PDB structures but, are absent in SARS-CoV spike glycoprotein structures.

When chrysin binds with the target molecule, the sequences of interacting amino acids e.g. PHE 486, TYR 484 and THR 487 are same in five SARS CoV-2 spike proteins and different from SARS-CoV spike glycoprotein structures.

Hesperidin is a major flavonoid compound, present in orange and lemon fruits. 470–761 mg/L of Hesperidin is normally present in orange juice^31^. This phytochemical exhibit various medicinal properties. According to oral toxicity study of hesperidin, it can be concluded that this phytochemical can be safely used in herbal formulations with its LD_50_ value more than 2000 mg/kg^31^. This flavanone glycoside, has a long medicinal history in both Indian and Chinese herbal medications^32^. This phytochemical alone or in combination with other chemicals, is often used to treat various diseases.

Emodin is a polyphenol found in the roots, bark and leaves of several plants including aloe vera, cascara, rhubarb, senna etc. In traditional medicine, emodin has been used for cardiovascular diseases and osteoporosis. It has been suggested earlier that emodin can inhibit influenza A virus replication^33^ via several cell signaling pathways.

Chrysin a natural flavonoid, is commonly found in propolis and honey. As reported earlier, chrysin can act as an inhibitor during enterovirus 71 (EV71) growth and replication^34^. Similarly, Song et al.^35^described antiviral activity of chrysin against coxsackievirus B3 (CVB3).

Considering the results obtained from molecular docking studies, phytochemicals hesperidin, emodin and chrysin can be used for COVID-19 treatment, after in-silico mutagenesis study and experimental verification. These phytochemicals have shown comparable spike protein inhibiting efficacy as that of known inhibitors such as chloroquine and hydroxychloroquine. From the molecular dynamics and QSAR study, it can be concluded that for ACE2 receptor protein, ligand binding activity of spike protein fragment, will be decreased noncompetitively by modulator hesperidin. So, this natural compound can show antiviral activity by destabilizing spike protein binding with human host ACE2 receptor. The modulation of hesperidin of ACE2 protein try to prevent its interaction with spike protein. It has been proved by a simple in silico experiment and the result of this experiment in shown in Supplementary Information S3.

## Methodology

### Protein molecular modeling of spike protein fragment

The 3D structure of spike protein (from amino acid sequence 331 to 524) and its binding site with its host cell receptor ACE2 protein have been highlighted in this study. In RSCB database, the available 3D structure of spike protein (PDB ID 6LZG) contains two ligands e.g. Zn^2+^ and NAG. Only primary amino acid sequence of spike protein (from position 331 to 524) and its 3D structure, is relevant for our study. So, by using template sequence 6lzg.1. B with 100% sequence identity, for primary sequence (from position 331 to 524), modeled structure of spike protein fragment, has been created by homology modeling study. Homology modeled structure of RBD fragment (from amino acid residues 331 to 524 of spike protein) in SARS-CoV-2 is considered in this paper as responsible fragment for strong binding with ACE2 receptor protein of human. Before molecular docking analysis, following steps are performed with the primary sequence of spike protein fragment using homology modeling technique.

#### Retrieval of protein sequence for spike protein fragment

The protein sequence of spike glycoprotein from Severe Acute Respiratory Syndrome Coronavirus 2 (SARS-CoV2) containing 193 amino acid residues from positions 331 to 524 is retrieved from GenBank database (https://www.ncbi.nlm.nih.gov/protein/QHR63250.2) in FASTA format and considered as spike protein fragment in this study.

#### 3D structure homology modeling and validation of modeled structure

In modeling 3D structure of the spike protein fragment by using sequence homology approach, first of all sequence alignment method is used. Thus, the best matching PDB structures of other proteins are identified with the help of following steps:

##### Template Search for the spike protein fragment

Template search for homology modeling of protein with Blast^22^ and HHBlits^23^ has been made against the SWISS-MODEL template library (SMTL, last updated on 2020-04-08 and last included in PDB release on 2020-04-03). A total of 63 templates for the target sequence are found with Blast^22^. Similarly, a total of 110 templates are found for HHBlits^23^.

##### Template selection for the spike protein fragment

Only the templates with the highest quality have been selected from the features of the target-template alignment for model building of spike protein fragment.

##### Model building for the spike protein fragment

Models are constructed based on the target-template alignment by ProMod3. In case, loop modelling with ProMod3 fails, an alternative model is made with PROMOD-II^36^.

##### Model quality estimation for the spike protein fragment

The best model among obtained models by using two types of selection methods are estimated by QMEAN4 scores^37^ and Ramachandran plot^38,39^. The Ramachandran plots for two models are obtained using PROCHECK^38^ and MolProbity^39^. Evaluation of backbone conformation of protein molecule is assayed by Ramachandran plot dividing the percentage of amino acid residues of the model in the allowed and disallowed regions^38,39^.

### Molecular docking between S protein fragment and ACE2

Molecular docking studies between S protein fragment and human ACE2 receptor are performed using ClusPro^27^. Following equation has been used to compute cluster scores as well as to predict the lowest binding energy (using ClusPro 2.2 online server^27^) –

$${\text{E}} = 0.40{\text{E}}_{{{\text{rep}}}} +{\ {   - 0}}.40{\text{E}}_{\text{att}} + 600\text{E} _{{{\text{elec}}}}  + 1.00{\text{E}}_{\text{DARS}}.$$

The repulsive {rep}, attractive {att}, electrostatic {elec} forces and interactions extracted from the decoys as the reference state {DARS}, are measured using molecular docking study^40^.

### Molecular docking study of phytochemicals obtained from Indian medicinal plants

Docking of bound structure (spike protein fragment and its receptor ACE2) with phytochemicals are executed with SWISSDOCK web server and constructed by EADock DSS^41^. Similarly, two known inhibitors of coronavirus spike proteins such as chloroquine and hydroxychloroquine are also used for similar docking studies with bound structure of ACE2 protein and spike protein fragment. Many binding modes are predicted in the vicinity of all target cavities, which is known as blind docking. The CHARMM energies are estimated on a grid using CHARMM force field^42^ from Swiss Institute of Bioinformatics.

The most favourable energies for the corresponding binding modes are evaluated with FACTS^43^ and are therefore clustered. Subsequently, the molecular complexes are ranked based on these energies. Among those complexes, the one structure, which represents the best binding mode, is selected for each phytochemical. The most favourable clusters are visualized by the USCF Chimera software^44,45^.

The protein–protein interaction energy between spike protein fragment and its receptor ACE2 is an important criterion to evaluate the effect of natural products, which could bind to the binding surface between spike protein fragment and ACE2 receptor. Binding affinity defined as the strength of protein–protein interaction, which is related with the cellular functions of those proteins. This binding energy can be expressed as a physiochemical parameter, known as dissociation constant (Kd)^46,47^. Prediction of binding affinity between spike protein fragment and ACE2 receptor following contact-based prediction of binding affinity method^46^ in protein–protein complexes, using PRODIGY web server^47^ has been performed.

### Binding affinity calculation in different docking structures

The strength of protein–ligand binding is known as binding affinity. The attraction force between the bound structure of S protein fragment, ACE2 and Hesperidin determine the binding affinity of the ligand towards protein. This affinity determines whether a ligand finally will bound or separate from the protein surface and return to its unbound state. The binding affinity of different docking structures in absence and presence of spike protein for non-competitive modulators are calculated using Dockthor web server^48^.

## Supplementary information

Supplementary Information 1

Supplementary Information 2

Supplementary Information 3

## Data Availability

All data generated or analyzed during this study are included in this published article (and its Supplementary Information files).
