# Supplementary material for: Molecular docking study of potential phytochemicals and their effects on the complex of SARS-CoV2 spike protein and human ACE2
Source: Sci Rep. 2020 Oct 19;10:17699. doi: 10.1038/s41598-020-74715-4 (PMC7573581; doi:10.1038/s41598-020-74715-4)
Supplement: Supplementary file 1 — Supplementary Information 1 [file 41598_2020_74715_MOESM1_ESM.pdf]

Supplementary File S1

Molecular docking study of potential phytochemicals and their effects on the complex of SARS-CoV2 spike protein and human ACE2

Anamika Basu[1], Anasua Sarkar[2,*], Ujjwal Maulik[2]

[1] *Department of Biochemistry, Gurudas College, India, Email: basuanamikaami@gmail.com*

[2] *Computer Science and Engineering Department, Jadavpur University, India, Email: anasua.sarkar@jadavpuruniversity.in*

[3] *Computer Science and Engineering Department, Jadavpur University, India, Email: umaulik@cse.jdvu.ac.in*

I. Detailed analysis of intermolecular contacts (ICs) and the list of amino acidswhich are present in the protein -protein interaction sites of spike protein fragment and ACE2 receptor in absence and presence of phytochemicals

Predicting binding affinity from structural models of protein-protein interaction, plays an important role in drug design. Comparative study for protein-protein binding interaction energy values as obtained in our research work, according to PRODIGY web server, is shown in the following table (Table S1.1):

Table S1.1 Comparative study for protein-protein binding interaction energy values

**Features**	**Compounds**	
	**Spike protein fragment bound with ACE2 receptor**	**Spike protein fragment bound with ACE2 receptor in presence of hesperidin**
ΔG (binding energy) Kcal/mole	15.4	14.5
K_d (dissociation constant) at 25°C	$5.1E^{-12}$	$2.4E^{-11}$
Intermolecular contacts (ICs) at the interface between the two protein within the threshold distance of 5Å	ICs charged-charged: 5<br>ICs charged-polar: 10<br>ICs charged-apolar: 19<br>ICs polar-polar: 9<br>ICs polar-apolar: 40<br>ICs apolar-apolar: 18	ICs charged-charged: 3<br>ICs charged-polar: 7<br>ICs charged-apolar: 15<br>ICs polar-polar: 6<br>ICs polar-apolar: 36<br>ICs apolar-apolar: 12

The interface between spike protein fragment and ACE2 receptor in absence of natural product is shown in following Figure S1.1.

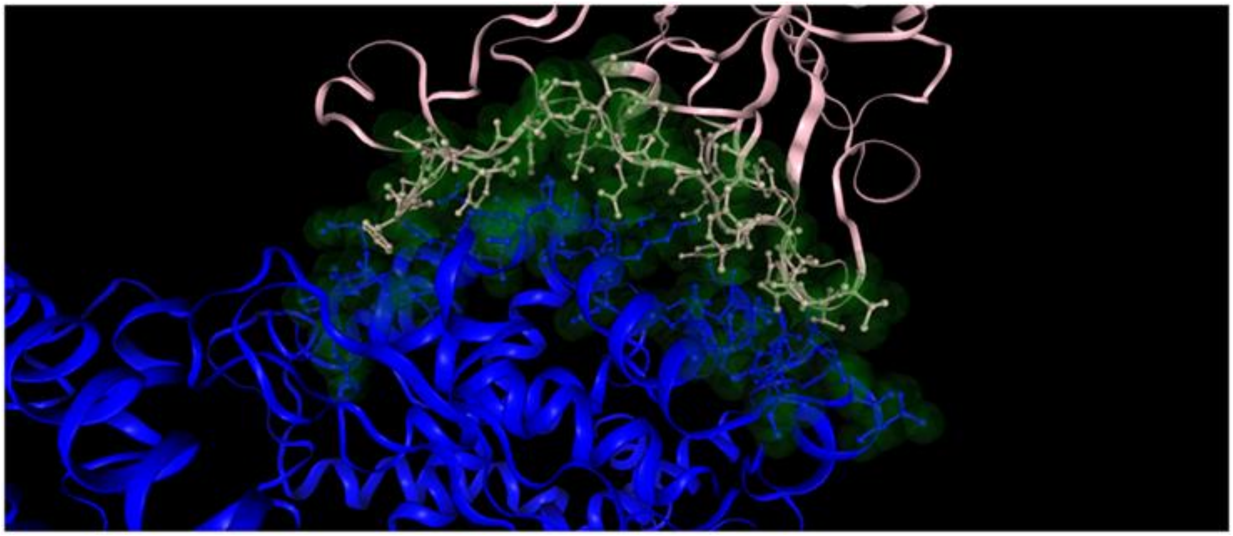

Figure S1.1 The interface between spike protein fragment and ACE2 receptor in absence of natural product.

Interacting chains are colored in blue and pink (ACE2 and spike protein fragment respectively). Residues which are present at the interface, are highlighted with green spheres.

Table S1.2 List of amino acids are present in the protein-protein interaction sites of spike protein fragment with ACE2 receptor.

PRO	426	A	THR	158	B
SER	317	A	TYR	111	B
ARG	306	A	TYR	107	B
LYS	313	A	PHE	155	B
PRO	321	A	PHE	144	B
PRO	426	A	GLY	160	B
VAL	316	A	LEU	150	B
PRO	321	A	GLY	143	B
GLU	310	A	GLY	104	B
THR	548	A	LYS	75	B
GLN	552	A	ASN	145	B
SER	425	A	THR	158	B
VAL	316	A	GLN	151	B
ASN	322	A	GLU	142	B
TYR	385	A	PHE	144	B
ARG	559	A	ASN	145	B
SER	420	A	ASN	159	B
LEU	320	A	PHE	114	B
LYS	313	A	GLN	151	B
LYS	419	A	THR	158	B
ALA	384	A	PHE	144	B
GLN	300	A	GLN	156	B
LYS	416	A	TYR	163	B
GLY	422	A	GLN	156	B
ILE	421	A	GLN	156	B
LYS	313	A	TYR	153	B
LYS	313	A	TYR	107	B

ASN	546	A	LEU	113	B
LEU	558	A	PHE	144	B
MET	383	A	PHE	144	B
ARG	559	A	PHE	144	B
ASP	299	A	VAL	103	B
ASN	322	A	PHE	148	B
VAL	316	A	PHE	114	B
PHE	555	A	PHE	144	B
LYS	419	A	GLY	160	B
GLN	552	A	ALA	133	B
SER	317	A	GLN	151	B
ASN	546	A	LYS	75	B
PHE	555	A	ASN	145	B
PRO	321	A	CYS	146	B
LEU	424	A	THR	158	B
LEU	423	A	THR	158	B
THR	548	A	LEU	113	B
GLY	422	A	THR	158	B
VAL	318	A	LEU	113	B
TRP	302	A	GLN	156	B
PRO	321	A	GLU	142	B
ARG	306	A	GLY	104	B
LYS	419	A	ASN	159	B
LEU	423	A	GLN	156	B
GLY	422	A	ASN	159	B
PHE	555	A	TYR	147	B
GLN	300	A	THR	158	B
ILE	421	A	ASN	159	B
GLU	310	A	TYR	107	B
GLY	319	A	PHE	114	B
PHE	314	A	GLN	151	B
LYS	313	A	SER	152	B
ASN	546	A	TYR	163	B
THR	548	A	PHE	114	B
LEU	560	A	PHE	144	B
ASN	546	A	TYR	111	B
ARG	306	A	GLY	105	B
TRP	302	A	GLY	104	B
PRO	426	A	ASN	159	B
VAL	316	A	LEU	113	B
SER	420	A	GLY	160	B
SER	317	A	LEU	113	B
GLY	319	A	TYR	147	B
LYS	313	A	GLY	154	B
SER	420	A	GLY	154	B
PRO	321	A	TYR	147	B
GLU	310	A	GLN	156	B
ARG	306	A	GLN	156	B
LEU	424	A	ASN	159	B
VAL	318	A	PHE	114	B
GLY	319	A	LEU	113	B
MET	383	A	GLY	143	B
VAL	316	A	PHE	148	B
GLN	300	A	GLY	104	B
GLN	300	A	VAL	103	B
ASN	546	A	ARG	61	B
ILE	421	A	GLY	154	B

SER	420	A	TYR	163	B
LEU	320	A	TYR	147	B

The interface between spike protein fragment and ACE2 receptor in presence of hesperidin is shown in following Figure S1.2.

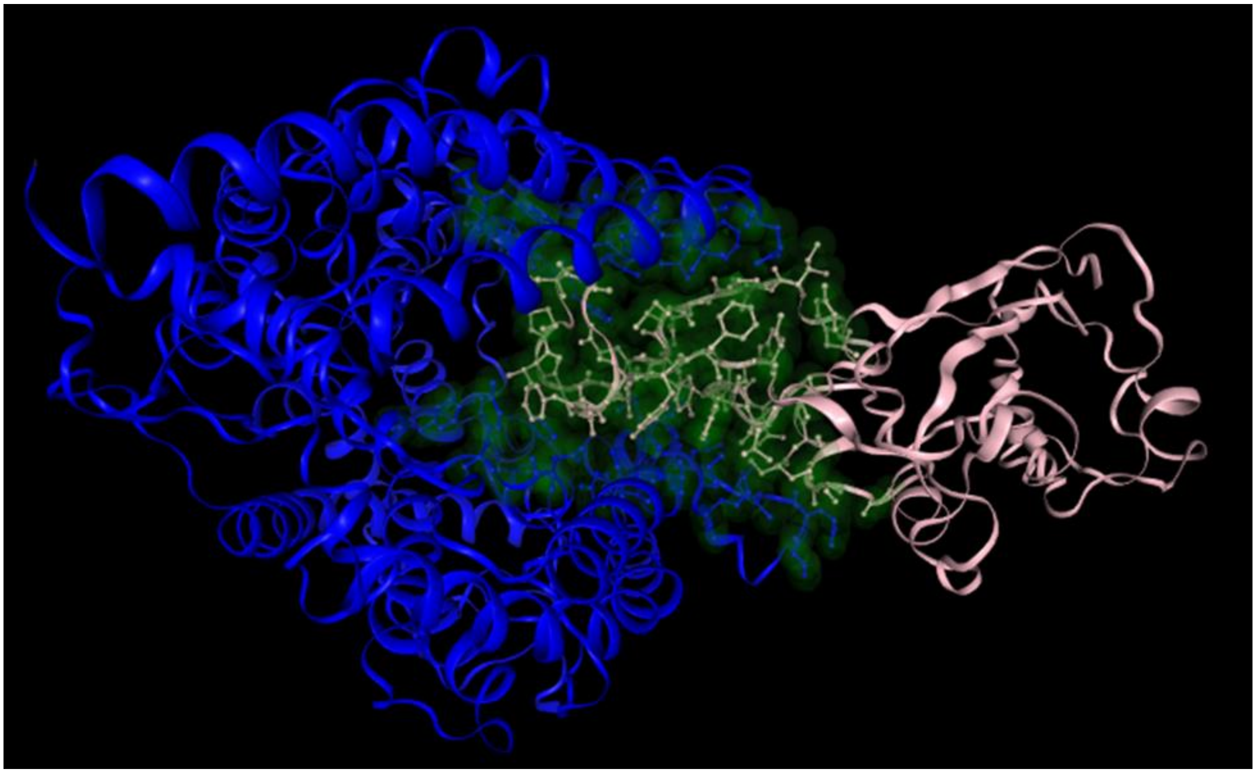

Figure S1.2 The interface between spike protein fragment and ACE2 receptor in presence of hesperidin.

Interacting chains are colored in blue and pink (ACE2 and spike protein fragment respectively). Residues which are present at the interface, are highlighted with green spheres.

Table S1.3 List of amino acids which are present in the protein-protein interaction sites of spike protein fragment with ACE2 receptor in presence of hesperidin.

VAL	59	A	ILE	130	B
SER	128	A	TYR	131	B
LEU	143	A	TYR	79	B
PRO	138	A	THR	73	B
LEU	143	A	ARG	115	B
VAL	343	A	ILE	130	B
LEU	143	A	LYS	116	B
LEU	143	A	TYR	131	B
TRP	349	A	ASN	139	B

TYR	127	A	ASN	145	B
PRO	146	A	LYS	116	B
TYR	510	A	PHE	144	B
ASN	51	A	ASN	139	B
GLU	56	A	LEU	150	B
LYS	341	A	THR	128	B
SER	124	A	ASN	145	B
SER	47	A	GLY	140	B
ASN	508	A	TYR	147	B
LYS	131	A	ASN	118	B
THR	129	A	TYR	79	B
TYR	50	A	THR	128	B
MET	62	A	GLY	140	B
PRO	146	A	SER	117	B
SER	124	A	PHE	144	B
GLU	145	A	LYS	116	B
TRP	349	A	GLY	140	B
ASN	508	A	GLY	143	B
MET	62	A	ILE	130	B
SER	128	A	TYR	147	B
THR	129	A	LYS	75	B
HIS	505	A	THR	136	B
GLU	56	A	PHE	148	B
SER	128	A	ALA	133	B
TYR	50	A	GLU	129	B
ILE	54	A	GLU	129	B
ILE	54	A	THR	128	B
THR	125	A	TYR	147	B
THR	347	A	ASN	139	B
GLU	145	A	GLY	134	B
LYS	341	A	GLU	129	B
SER	124	A	TYR	147	B
GLN	139	A	THR	73	B
GLU	145	A	ALA	133	B
CYS	141	A	ASN	118	B
THR	129	A	LEU	113	B
THR	129	A	TYR	131	B
LYS	131	A	ASP	78	B
THR	125	A	PHE	114	B
ASN	508	A	PHE	144	B
THR	347	A	PRO	137	B
VAL	59	A	THR	128	B
PHE	504	A	THR	136	B
SER	124	A	GLY	143	B
LYS	341	A	SER	127	B
ALA	348	A	ASN	139	B
THR	129	A	PHE	114	B
GLU	56	A	THR	128	B
LYS	341	A	ILE	126	B
SER	128	A	PHE	114	B
PHE	504	A	SER	135	B
HIS	345	A	PRO	137	B
VAL	343	A	GLU	129	B
GLN	139	A	GLY	71	B
PHE	504	A	ASN	145	B
GLU	56	A	TYR	9	B
GLU	56	A	ILE	126	B

SER	47	A	ASN	139	B
LYS	131	A	TYR	79	B
PRO	346	A	PRO	137	B
ASN	508	A	ASN	145	B
ASN	51	A	GLY	140	B
LEU	143	A	ASN	118	B
TYR	515	A	THR	136	B
HIS	345	A	GLN	132	B
CYS	141	A	THR	73	B
VAL	59	A	PHE	148	B
PHE	504	A	PHE	144	B
TYR	50	A	ILE	130	B
SER	124	A	CYS	146	B
