# Supplementary material for: Molecular docking study of potential phytochemicals and their effects on the complex of SARS-CoV2 spike protein and human ACE2
Source: Sci Rep. 2020 Oct 19;10:17699. doi: 10.1038/s41598-020-74715-4 (PMC7573581; doi:10.1038/s41598-020-74715-4)
Supplement: Supplementary file 2 — Supplementary Information 2 [file 41598_2020_74715_MOESM2_ESM.pdf]

Molecular docking study of potential phytochemicals and their effects on the complex of SARS-CoV2 spike protein and human ACE2

Anamika Basu[1], Anasua Sarkar[2,*], Ujjwal Maulik[3]

[1] *Department of Biochemistry, Gurudas College, India, Email: basuanamikaami@gmail.com*

[2] *Computer Science and Engineering Department, Jadavpur University, India, Email: anasua.sarkar@jadavpuruniversity.in*

[3] *Computer Science and Engineering Department, Jadavpur University, India, Email: umaulik@cse.jdvu.ac.in*

Supplementary File S2

1. QSAR study to identify anti-viral activity of five natural compounds

Quantitative Structure–Activity Relationship (QSAR) analysis is used to identify anti-viral activities of different approved anti-viral drugs, which are experimentally studied against SARS CoV 2. The experimental validation of the anti-viral activity of chosen five natural compounds and their ability to inhibit the S protein-ACE2 interaction is shown here by QSAR study. In this QSAR study of various antiviral compounds, some descriptor parameters of selected chemical properties with calculated binding energies with ACE2 & spike protein structure, are used to correlate with quantitative structure-activity relationship (QSAR) analysis using multiple linear regression (MLR) model.

In various models developed using MLR technique, the statistical parameters, like cross validated coefficient (CV) defines the goodness of prediction, whereas the non-cross validated conventional correlation coefficient (r^2) defines the goodness of fit of the QSAR model.

There are several steps in QSAR model development. First a dataset of similar chemical compounds with same biological activity have been identified. This QSAR study is performed with 43 compounds. First 36 compounds [1], [2], [3], [4], [5], [6] and their antiviral activities are compared with chosen five natural compounds and two well-known drugs for treatment of COVID-19 (chloroquine and hydroxychloroquine) [7], [8] with inhibitory effects against SARS CoV 2 in this study. Then different types of descriptors are calculated and using generic algorithm most suitable descriptors have been identified. With the help of these selected descriptors QSAR model is constructed.

The model is generated using four descriptors in Multiple Linear Regression model in BuildQSAR software [9]. The QSAR Model is represented as QSAR equation, with the correlation coefficients calculated for each descriptor used in the regression model. By plotting the experimental activity (Yobs) vs predicted activity (Ycalc), the built model is evaluated for its predictive powers to determine activity as QSAR model for anti-viral activity of five natural compounds. One linear model is built using four selected descriptors.

Table S2.1 Compound details for the QSAR study

Compound number	ChEMBL ID	Drug Name	ACE2-RBD binding affinity scores	Smiles

1	CHEMBL1229211	DOLUTEGRAVIR	-9.2	C[C@@H]1CCO[C@H]2Cn3cc(C(=O)NCc4ccc(F)cc4F)c(=O)c(O)c3C(=O)N21
2	CHEMBL707	DOXAZOSIN	-9.5	COc1cc2nc(N3CCN(C(=O)C4COc5ccccc5O4)CC3)nc(N)c2cc1OC
3	CHEMBL3301620	TEMSAVIR	-9.3	COc1cnc(-n2cnc(C)n2)c2[nH]cc(C(=O)C(=O)N3CCN(C(=O)c4ccccc4)CC3)c12
4	CHEMBL3989849	FENTONIUM	-9.2	C[N+]1(CC(=O)c2ccc(-c3ccccc3)cc2)[C@H]2CC[C@@H]1C[C@H](OC(=O)[C@H](CO)c1ccccc1)C2
5	CHEMBL254316	RALTEGRAVIR	-9.1	Cc1nnc(C(=O)NC(C)(C)c2nc(C(=O)NCc3ccc(F)cc3)c(O)c(=O)n2C)o1
6	CHEMBL3039520	LASMIDITAN	-9.1	CN1CCC(C(=O)c2cccc(NC(=O)c3c(F)cc(F)cc3F)n2)CC1
7	CHEMBL779	TADALAFIL	-9.1	CN1CC(=O)N2[C@H](Cc3c([nH]c4ccccc34)[C@H]2c2ccc3c(c2)OCO3)C1=O
8	CHEMBL1200376	BETAMETHASONE BENZOATE	-9	C[C@H]1C[C@H]2[C@@H]3CCC4=CC(=O)C=C[C@]4(C)[C@@]3(F)[C@@H](O)C[C@]2(C)[C@@]1(OC(=O)c1ccccc1)C(=O)CO
9	CHEMBL2403238	CABOTEGRAVIR	-9	C[C@H]1CO[C@@H]2Cn3cc(C(=O)NCc4ccc(F)cc4F)c(=O)c([O-])c3C(=O)N12.[Na+]
10	CHEMBL85	RISPERIDONE	-9	Cc1nc2n(c(=O)c1CCN1CCC(c3noc4cc(F)ccc34)CC1)CCCC2
11	CHEMBL9967	PIRENZEPINE	-8.7	CN1CCN(CC(=O)N2c3ccccc3C(=O)Nc3cccnc32)CC1
12	CHEMBL1481	GLIMEPIRIDE	-8.6	CCC1=C(C)CN(C(=O)NCCc2ccc(S(=O)(=O)NC(=O)N[C@H]3CC[C@H](C)CC3)cc2)C1=O
13	CHEMBL408	TROGLITAZONE	-8.6	Cc1c(C)c2c(c(C)c1O)CCC(C)(COc1ccc(CC3SC(=O)NC3=O)cc1)O2
14	CHEMBL428880	CROMOLYN	-8.6	O=C(O)c1cc(=O)c2c(OCC(O)COc3cccc4oc(C(=O)O)cc(=O)c34)cccc2o1
15	CHEMBL121883	ZINVIROXIME	-8.5	CC(C)S(=O)(=O)n1c(N)nc2ccc(/C(=N\O)c3ccccc3)cc21
16	CHEMBL237500	LINAGLIPTIN	-8.4	CC#CCn1c(N2CCC[C@@H](N)C2)nc2c1c(=O)n(Cc1nc(C)c3ccccc3n1)c(=O)n2C
17	CHEMBL4297592	AMENAMEVIR	-8.4	Cc1cccc(C)c1N(CC(=O)Nc1ccc(-c2ncon2)cc1)C(=O)C1CCS(=O)(=O)CC1
18	CHEMBL1512	FLUNISOLIDE	-8.2	CC1(C)O[C@@H]2C[C@H]3[C@@H]4C[C@H](F)C5=CC(=O)C=C[C@]5(C)[C@H]4[C@@H](O)C[C@]3(C)[C@]2(C(=O)CO)O1
19	CHEMBL2018096	IPRAGLIFLOZIN	-8.2	OC[C@H]1O[C@@H](c2ccc(F)c(Cc3cc4ccccc4s3)c2)[C@H](O)[C@@H](O)[C@@H]1O
20	CHEMBL330115	CHEMBL330115	-8.2	CC(C)(C)NC(=O)[C@H]1N(C(=O)[C@@H](O)[C@H](Cc2ccccc2)NC(=O)c2ccccc2)CSC1(C)C
21	CHEMBL1697771	REBAMIPIDE	-8	O=C(NC(Cc1cc(=O)[nH]c2ccccc12)C(=O)O)c1ccc(Cl)cc1
22	CHEMBL4297215	BALOXAVIR	-7.9	O=C1c2c(O)c(=O)ccn2N([C@@H]2c3ccccc3SCc3c2ccc(F)c3F)[C@@H]2COCCN12
23	CHEMBL1346	DARIFENACIN	-7.6	NC(=O)C(c1ccccc1)(c1ccccc1)[C@@H]1CCN(CCc2ccc3c(c2)CCO3)C1
24	CHEMBL1201187	MARAVIROC	-9.7	Cc1nnc(C(C)C)n1[C@H]1C[C@@H]2CC[C@H](C1)N2CC[C@H](NC(=O)C1CCC(F)(F)CC1)c1ccccc1
25	CHEMBL3695568	JNJ-49095397	-9.3	COCC(=O)Nc1cc(COc2ccc(NC(=O)Nc3cc(C(C)(C)C)nn3-c3ccc(C)cc3)c3ccccc23)ccn1

26	CHEMBL1257073	TECOVIRIMAT	-9.2	O=C(NN1C(=O)[C@H]2[C@H]3C=C[C@H]([C@@H]4C[C@H]34)[C@H]2C1=O)c1ccc(C(F)(F)F)cc1
27	CHEMBL4297402	VP-14637	-9.2	Cc1nnnn1N=Cc1ccc(O)c(C(c2ccc(O)cc2)c2cc(C=Nn3nnnc3C)ccc2O)c1
28	CHEMBL4297639	LORECIVIVINT	-9.2	CC(C)CC(=O)Nc1cncc(-c2ccc3[nH]nc(-c4nc5cncc(-c6cccc(F)c6)c5[nH]4)c3c2)c1
29	CHEMBL223402	RSV-604	-9	O=C(Nc1ccccc1F)N[C@H]1N=C(c2ccccc2)c2ccccc2NC1=O
30	CHEMBL1241951	LETERMOVIR	-8.7	COc1cccc(N2CCN(C3=Nc4c(F)cccc4[C@H](CC(=O)O)N3c3cc(C(F)(F)F)ccc3OC)CC2)c1
31	CHEMBL3039531	DANIRIXIN	-8.7	Cc1c(F)cccc1NC(=O)Nc1ccc(Cl)c(S(=O)(=O)[C@H]2CCCNC2)c1O
32	CHEMBL160	CYCLOSPORINE	-7.9	C/C=C/C[C@@H](C)[C@@H](O)[C@H]1C(=O)N[C@@H](CC)C(=O)N(C)CC(=O)N(C)[C@@H](CC(C)C)C(=O)N[C@@H](C(C)C)C(=O)N(C)[C@@H](CC(C)C)C(=O)N[C@@H](C)C(=O)N[C@H](C)C(=O)N(C)[C@@H](CC(C)C)C(=O)N(C)[C@@H](CC(C)C)C(=O)N(C)[C@@H](C(C)C)C(=O)N1C
33	CHEMBL64391	ITRACONAZOLE	-7.9	CCC(C)n1ncn(-c2ccc(N3CCN(c4ccc(OCC5COC(Cn6cncn6)(c6ccc(Cl)cc6Cl)O5)cc4)CC3)cc2)c1=O
34	CHEMBL204656	ELVITEGRAVIR	-7.2	COc1cc2c(cc1Cc1cccc(Cl)c1F)c(=O)c(C(=O)O)cn2[C@H](CO)C(C)C
35	CHEMBL4291143	PC-786	-12.1	Cc1cnc(N2CC3(CCOCC3)C2)c(C(=O)Nc2ccc(C(=O)N3CCc4cc(C(=O)Nc5c(C)cccc5F)sc4-c4ccccc43)cc2)c1
36	CHEMBL3601411	TEGAVIVINT	-10.2	C[C@H]1C[C@@H](C)CN(S(=O)(=O)c2ccc3c(c2)C(=NO)c2cc(S(=O)(=O)N4C[C@@H](C)C[C@@H](C)C4)ccc2C3=NO)C1
37	CHEMBL449317	HESPERIDIN	-8.99	COc1ccc([C@@H]2CC(=O)c3c(O)cc(O[C@@H]4O[C@H](CO[C@@H]5O[C@@H](C)[C@H](O)[C@@H](O)[C@H]5O)[C@@H](O)[C@H](O)[C@H]4O)cc3O2)cc1O
38	CHEMBL117	CHRYSIN	-6.87	O=c1cc(-c2ccccc2)oc2cc(O)cc(O)c12
39	CHEMBL55659	ANTHRAQUINONE	-6.15	O=C1c2ccccc2C(=O)c2ccccc21
40	CHEMBL289277	EMODIN	-6.19	Cc1cc(O)c2c(c1)C(=O)c1cc(O)cc(O)c1C2=O
41	CHEMBL418068	RHEIN	-8.73	O=C(O)c1cc(O)c2c(c1)C(=O)c1cccc(O)c1C2=O
42	CHEMBL76	CHLOROQUINE	-7.1	CCN(CC)CCCC(C)Nc1ccnc2cc(Cl)ccc12
43	CHEMBL1690	HYDROXYCHLOROQUINE	-6.8	CCN(CCCC(C)NC1=C2C=CC(=CC2=NC=C1)Cl)CCO

Using QSAR study, the binding energy of these antiviral compounds with ACE2 and spike protein are computed. The corresponding linear regression equation for the QSAR model is

Binding energy = - 0.6785 (± 0.5614) AWeight + 1.2212 (± 0.5594) ncocl - 3.0467 (± 0.8601) diametert + 0.1178 (± 0.0551) Bertzct + 11.3538 (± 8.3667)

($n = 43$; $R = 0.801$; $s = 0.664$; $F = 17.051$; $p < 0.0001$; $Q2 = 0.530$; SPress = 0.761 ; SDEP = 0.724)

This equation is obtained by analyzing the two constitutional descriptors, namely - Average molecular weight (not including H) descriptor, Count of Cl atoms descriptor and two topological

descriptors, namely - Largest value in the distance matrix descriptor and BertzCT descriptor (complexity index, taking into account both the variety of kinds of bond connectivities and atom types). Here n is number of molecules under analysis, R is the correlation coefficient, r^2 (R) is the squared correlation coefficient, s is the standard deviation and F is the F statistical value. The cross validated squared correlation coefficient, Q2 is 0.530 and standard deviation of sum of square of difference between predicted and observed values, SPress is 0.761.

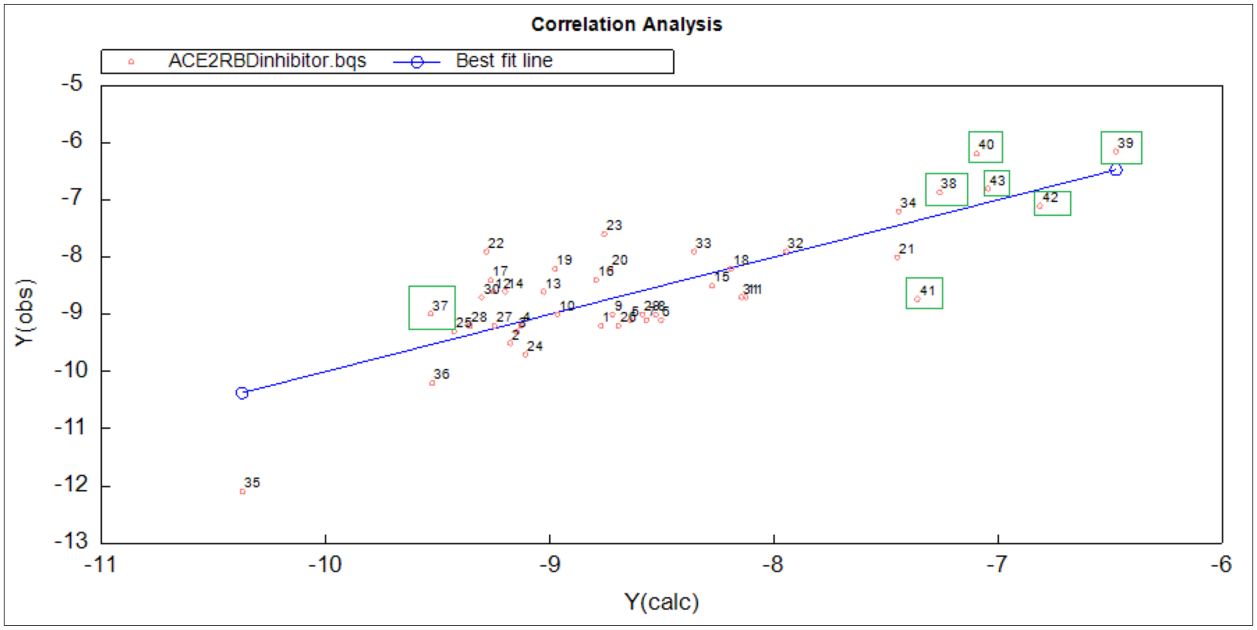

Figure S2.1 Scatter plot of Observed binding energy and calculated binding energy from experimented QSAR study

The plot for correlation analysis between observed binding energy and calculated binding energy, is shown in Figure S2.1. The observed activity shows linear relationship with calculated activity, because the corresponding data is well-fitted with the linear regression model. Higher value of r2(R) signifies that the relationship has smaller differences between the observed data and the fitted values.

Among chosen five natural compounds, four phytochemicals (marked as 37, 38, 39, 40) except rhein (marked as 41), show linear relationship with observed binding energy from molecular docking study and calculated binding energy from QSAR study. Same result is also observed for two known inhibitors, namely chloroquine and hydroxychloroquine (marked as 42 and 43) of spike protein.

QSAR Equation
=============

Binding energy = - 0.6785 (± 0.5614) AWeight + 1.2212 (± 0.5594) ncocl - 3.0467 (± 0.8601) diametert + 0.1178 (± 0.0551) Bertzct + 11.3538 (± 8.3667)

(n = 43 ; R = 0.801 ; s = 0.664 ; F = 17.051 ; p < 0.0001 ; Q2 = 0.530 ; SPress = 0.761 ; SDEP = 0.724)

Dataset

=======

No.	Compounds	Binding energy	AWeight	ncocl	diametert	Bertzct
1	CHEMBL1229211	-9.200	13.341	0.000	4.058	10.944
2	CHEMBL707	-9.500	12.918	0.000	4.232	9.583
3	CHEMBL3301620	-9.300	12.866	0.000	4.277	10.694
4	CHEMBL3989849	-9.200	12.510	0.000	4.340	10.424
5	CHEMBL254316	-9.100	13.227	0.000	4.121	13.035
6	CHEMBL3039520	-9.100	13.305	0.000	3.936	9.861
7	CHEMBL779	-9.100	12.768	0.000	3.993	7.694
8	CHEMBL1200376	-9.000	12.870	0.000	4.191	13.750
9	CHEMBL2403238	-9.000	13.387	0.000	4.021	10.694
10	CHEMBL85	-9.000	12.776	0.000	4.142	8.222
11	CHEMBL9967	-8.700	12.702	0.000	3.855	7.500
12	CHEMBL1481	-8.600	13.422	0.000	4.274	12.924
13	CHEMBL408	-8.600	13.366	0.000	4.149	11.285
14	CHEMBL428880	-8.600	13.301	0.000	4.246	11.944
15	CHEMBL121883	-8.500	13.611	0.000	3.777	9.451
16	CHEMBL237500	-8.400	12.695	0.000	4.223	11.306
17	CHEMBL4297592	-8.400	13.422	0.000	4.220	11.424
18	CHEMBL1512	-8.200	13.008	0.000	4.001	12.500
19	CHEMBL2018096	-8.200	13.689	0.000	3.981	9.222
20	CHEMBL330115	-8.200	13.211	0.000	4.186	13.875
21	CHEMBL1697771	-8.000	13.680	1.000	3.874	9.000
22	CHEMBL4297215	-7.900	13.657	0.000	4.125	10.167
23	CHEMBL1346	-7.600	12.385	0.000	4.160	8.201
24	CHEMBL1201187	-9.700	12.766	0.000	4.333	11.896
25	CHEMBL3695568	-9.300	12.646	0.000	4.556	14.257
26	CHEMBL1257073	-9.200	13.378	0.000	3.964	9.396
27	CHEMBL4297402	-9.200	12.851	0.000	4.342	11.444
28	CHEMBL4297639	-9.200	12.668	0.000	4.385	10.556
29	CHEMBL223402	-9.000	12.802	0.000	4.013	8.250
30	CHEMBL1241951	-8.700	13.276	0.000	4.370	14.118
31	CHEMBL3039531	-8.700	14.508	1.000	3.997	11.062
32	CHEMBL160	-7.900	12.832	0.000	5.149	43.250
33	CHEMBL64391	-7.900	13.619	2.000	4.769	13.729
34	CHEMBL204656	-7.200	13.700	1.000	4.010	12.694
35	CHEMBL4291143	-12.100	13.030	0.000	4.802	14.812
36	CHEMBL3601411	-10.200	13.812	0.000	4.364	15.181
37	CHEMBL449317	-8.990	13.402	0.000	4.503	16.361
38	CHEMBL117	-6.870	12.851	0.000	3.481	6.028
39	CHEMBL55659	-6.150	12.509	0.000	3.246	4.667
40	CHEMBL289277	-6.190	13.008	0.000	3.472	8.111
41	CHEMBL418068	-8.730	13.150	0.000	3.537	8.361
42	CHEMBL76	-7.100	13.349	1.000	3.687	7.667
43	CHEMBL1690	-6.800	13.464	1.000	3.747	7.917

Coefficient Analysis
====================

Predictor	Coef.	Stdev	95% Conf.	t-ratio	p	Comment
Constant	11.3538	4.0973	8.3667	2.7710	0.0085	
AWeight	-0.6785	0.2749	0.5614	-2.4678	0.0181	
ncocl	1.2212	0.2740	0.5594	4.4573	0.0001	
diametert	-3.0467	0.4212	0.8601	-7.2331	0.0000	
Bertzct	0.1178	0.0270	0.0551	4.3642	0.0001	

Correlation Matrix
==================

	AWeight	ncocl	diametert	Bertzct
AWeight	1	0.489	0.055	0.024
ncocl	0.489	1	0.003	0.045
diametert	0.055	0.003	1	0.726
Bertzct	0.024	0.045	0.726	1

Residual table
==============

No.	Compounds	Y(obs)	Y(calc)	Y(res)	StDev.Res	Comment
1	CHEMBL1229211	-9.200	-8.772	-0.428	-0.645	
2	CHEMBL707	-9.500	-9.175	-0.325	-0.489	
3	CHEMBL3301620	-9.300	-9.146	-0.154	-0.232	
4	CHEMBL3989849	-9.200	-9.128	-0.072	-0.108	
5	CHEMBL254316	-9.100	-8.640	-0.460	-0.693	
6	CHEMBL3039520	-9.100	-8.503	-0.597	-0.899	
7	CHEMBL779	-9.100	-8.568	-0.532	-0.802	
8	CHEMBL1200376	-9.000	-8.527	-0.473	-0.713	
9	CHEMBL2403238	-9.000	-8.720	-0.280	-0.422	
10	CHEMBL85	-9.000	-8.965	-0.035	-0.053	
11	CHEMBL9967	-8.700	-8.125	-0.575	-0.865	
12	CHEMBL1481	-8.600	-9.251	0.651	0.981	
13	CHEMBL408	-8.600	-9.026	0.426	0.641	
14	CHEMBL428880	-8.600	-9.199	0.599	0.903	
15	CHEMBL121883	-8.500	-8.275	-0.225	-0.339	
16	CHEMBL237500	-8.400	-8.793	0.393	0.593	
17	CHEMBL4297592	-8.400	-9.264	0.864	1.301	
18	CHEMBL1512	-8.200	-8.189	-0.011	-0.017	
19	CHEMBL2018096	-8.200	-8.976	0.776	1.169	
20	CHEMBL330115	-8.200	-8.728	0.528	0.795	
21	CHEMBL1697771	-8.000	-7.449	-0.551	-0.830	
22	CHEMBL4297215	-7.900	-9.282	1.382	2.081	Outlier
23	CHEMBL1346	-7.600	-8.757	1.157	1.743	
24	CHEMBL1201187	-9.700	-9.107	-0.593	-0.893	
25	CHEMBL3695568	-9.300	-9.427	0.127	0.191	
26	CHEMBL1257073	-9.200	-8.693	-0.507	-0.764	
27	CHEMBL4297402	-9.200	-9.246	0.046	0.069	
28	CHEMBL4297639	-9.200	-9.357	0.157	0.237	
29	CHEMBL223402	-9.000	-8.586	-0.414	-0.623	
30	CHEMBL1241951	-8.700	-9.304	0.604	0.910	
31	CHEMBL3039531	-8.700	-8.143	-0.557	-0.840	
32	CHEMBL160	-7.900	-7.944	0.044	0.066	
33	CHEMBL64391	-7.900	-8.356	0.456	0.687	
34	CHEMBL204656	-7.200	-7.442	0.242	0.364	
35	CHEMBL4291143	-12.100	-10.372	-1.728	-2.603	Outlier
36	CHEMBL3601411	-10.200	-9.524	-0.676	-1.018	
37	CHEMBL449317	-8.990	-9.531	0.541	0.814	
38	CHEMBL117	-6.870	-7.260	0.390	0.588	
39	CHEMBL55659	-6.150	-6.473	0.323	0.486	
40	CHEMBL289277	-6.190	-7.094	0.904	1.362	
41	CHEMBL418068	-8.730	-7.359	-1.371	-2.065	Outlier

42	CHEMBL76	-7.100	-6.812	-0.288	-0.434
43	CHEMBL1690	-6.800	-7.043	0.243	0.366

Analysis of Variance
====================

Source	DF	SS	MS	F	p
Regression	4	30.0643	7.5161	17.0507	0.0000
Error	38	16.7507	0.4408		
Total	42	46.8150			

Fitting Parameters
==================

Property	Value
n	43
k	4
R^2	0.6422
R^2-Adj.	0.6045
s	0.6639
F	17.0507
p	0.0000
Q2	0.5296
SPress	0.7613
SDEP	0.7241
C.V.	-7.7468

This QSAR investigation indicates that the descriptors, namely AWeight, ncocl, diametert and Bertzct, are chosen GA-selected descriptors for the set of anti-viral compounds. These chosen compounds can act as spike protein inhibitors for ACE2 binding in laboratory experimental studies and are found to have a great deal to positively contribute to biological activity - antiviral activity against SARS CoV2.

2. Molecular dynamics simulation study of bound structure of ACE2 & spike protein fragment

Molecular dynamics study with the bound structure of ACE2 & spike protein fragment is performed using simulation tool (http://mmb.irbbarcelona.org/MDWeb/index.php) [10], known as course-grained molecular dynamics (Brownian dynamics: C-alpha) after preparing the molecule with GROMACS molecular simulation and AMBER-99SB* forcefield is carried out. Simulation study is completed for 100 ps (Δt 0.01ps) with force constant 40.0 Kcal/mol*$Å^2$. Output frequency is recorded for 10 steps and the distance between alpha carbon atoms is 3.8 Å. The bound structures of ACE2 & spike protein fragment in absence and presence of hesperidin after molecular dynamics simulation study are compared here.

The bound structure of ACE2 & spike protein fragment in absence and presence of hesperidin after molecular dynamics simulation study is presented in Figure S2.2 (a) and (b). Structure of ACE2 protein molecules with spike protein fragment present in molecular dynamics trajectory is represented by ribbon structure in absence of hesperidin and by liquorice structure in presence of hesperidin.

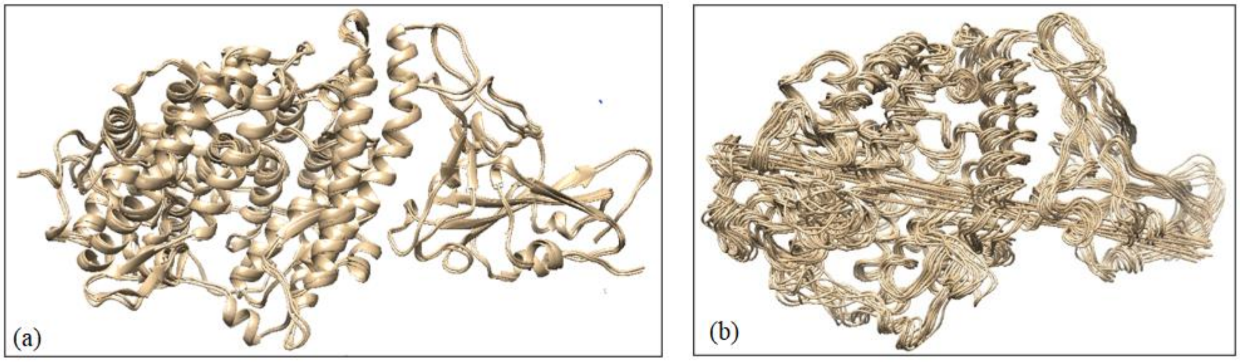

Figure S2.2 The bound structure of ACE2 & spike protein fragment (a) in absence and (b) presence of hesperidin

2.1 The B factor or temperature factor measurement in the bound structure of ACE2 & spike protein fragment

Atomic fluctuation or B factor or Debye Waller or temperature factors which are evaluated for each atom during a simulation study reflects the degree of thermal mobility and static disorder of an atom in a molecule structure. B factor for each residue for ACE2 protein in absence and presence of hesperidin in simulation study is shown in the following Figure S.2.3 a) and b) respectively.

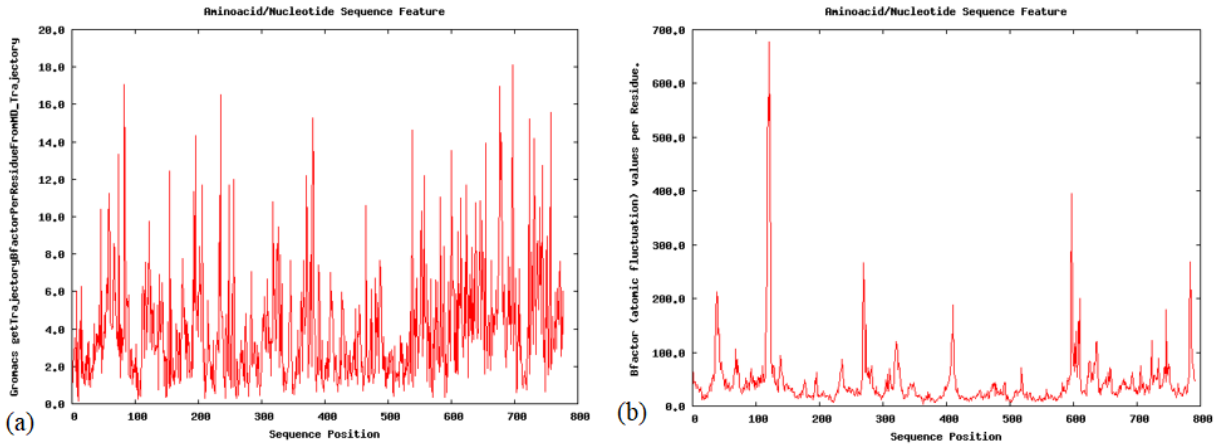

Figure S.2.3 B factor for each residue for ACE2 protein (a) in absence and (b) presence of hesperidin

In absence of hesperidin, B factor per residue varies from 0.0 to 20.0 and in presence of modulator the same characteristic changes from 0.0 to 700.0 for each residue of bound structure. Due to presence of hesperidin, the degree of thermal mobility and static disorder of atoms around 130 sequence position, are increased abruptly. This position of ACE2 protein is its ligand binding site for spike protein fragment (Figure 6 and 7). It proves that hesperidin can act as a modulator and decreases the ligand binding affinity of spike protein to its receptor ACE2 protein molecule.

2.2 The Radius of Gyration (Units in Angstrom) measurement in the bound structure of ACE2 & spike protein fragment

The radius of gyration is a quantitative measurement of the overall size of a protein molecule. The change in the structure of a protein during MD simulations can be quantified by radius of gyration. The radius of gyration of the bound structure of ACE2 & spike protein fragment in absence and presence of hesperidin after molecular dynamics simulations are presented in the following Figure S.2.4 a) and b). The radius of gyration of the bound structure of ACE2 & spike protein fragment decreases up to 7 snapshots of MD simulation and in last three snapshots remains almost unchanged. In presence of hesperidin molecule, the value of the radius of gyration of the bound structure of ACE2 & spike protein fragment fluctuates during MD simulation due to instability in docking structure.

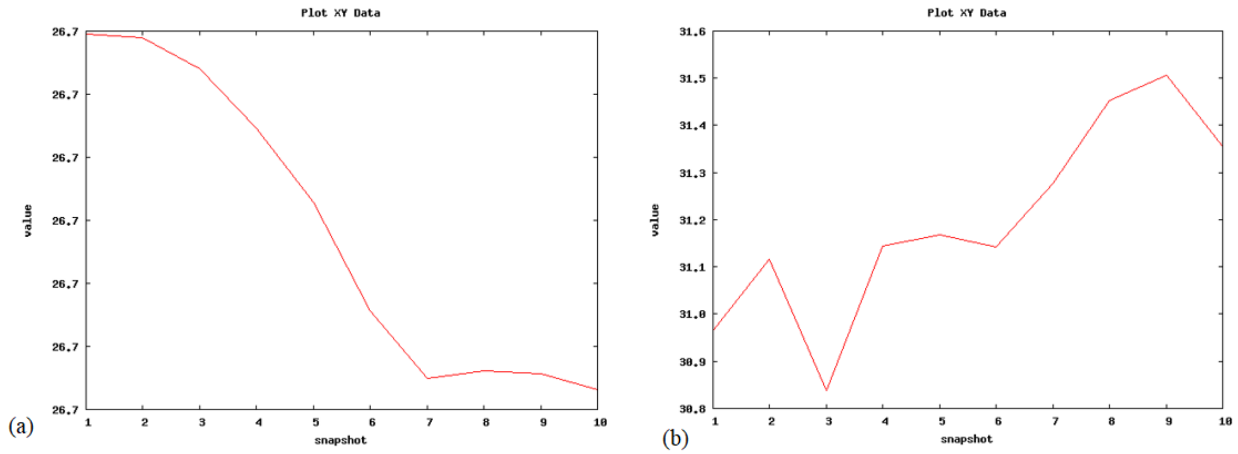

Figure S.2.4 The radius of gyration of the bound structure of ACE2 & spike protein fragment (a) in absence and (b) presence of hesperidin

2.3 Plot of RMSD along the trajectory

The root mean square deviation (RMSD) is an important tool that is used to characterize the conformational changes of proteins. The plot of RMSD of the bound structure of ACE2 & spike protein fragment in absence and presence of hesperidin after molecular dynamics simulations are presented in the following Figure S.2.5 a) and b). Comparing the nature of graph, it can be predicted that hesperidin causes conformational variation in docking structure.

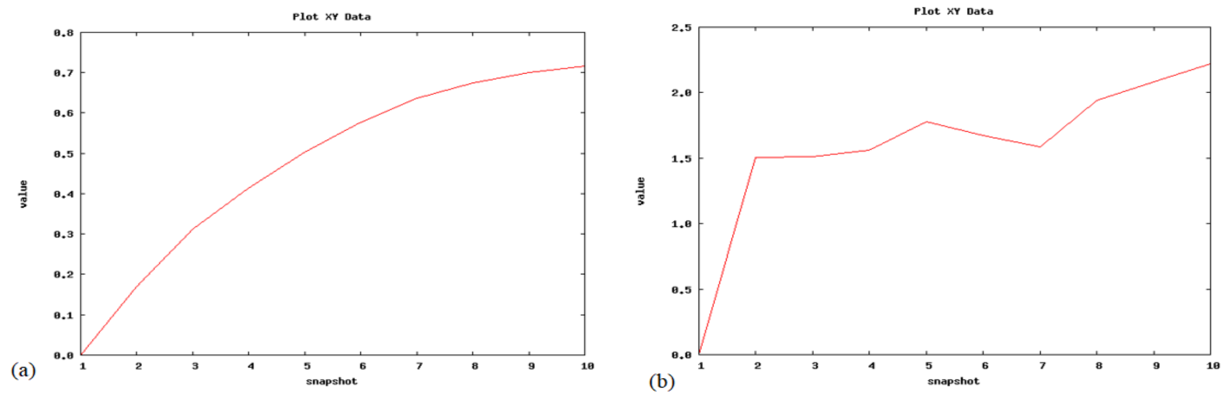

Figure S.2.5 Plot of RMSD along the trajectory (a) in absence and (b) presence of hesperidin

2.4 Plot of RMSD per residue of the bound structure of ACE2 & spike protein fragment

The root mean square deviation (RMSD) for each atom of the bound structure of ACE2 & spike protein fragment varies from 0.2Å to 0.8 Å during molecular simulation study (Figure S.2.6 (a)). This parameter increases up to the value of 5.0 Å in docking structure of ACE2 & spike protein due to the bonding of modulator molecule hesperidin (Figure S.2.6 (b)). This modulator molecule decreases the stability of the bound structure of ACE2 & spike protein fragment.

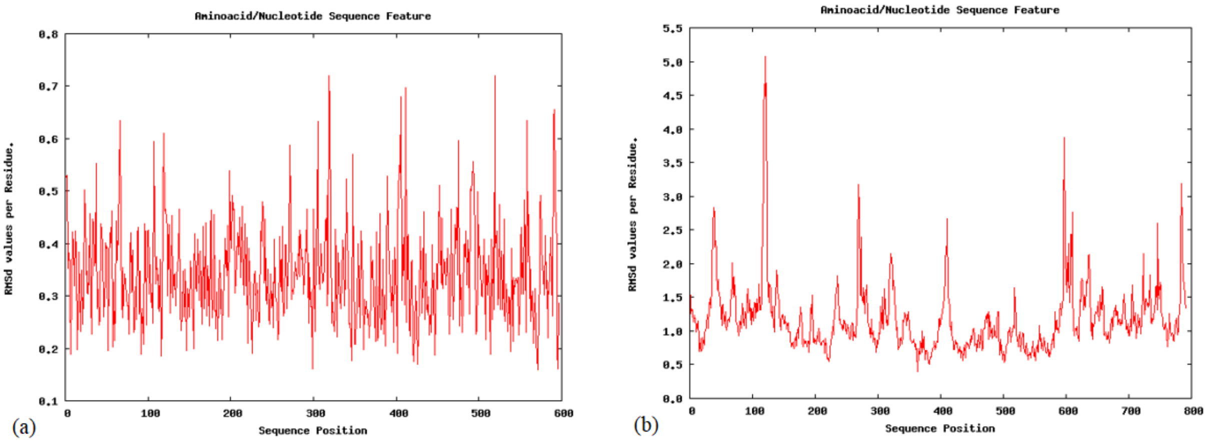

Figure S.2.6 Plot of RMSD per residue of the bound structure of ACE2 & spike protein fragment (a) in absence and (b) presence of hesperidin

From molecular simulation study for various parameters, it can be concluded that hesperidin can act as modulator for the bound structure of ACE2 & spike protein fragment. This analysis confirms the antiviral activity of natural compound hesperidin as non-competitive allosteric modulator of spike protein binding with its human host receptor.

REFERENCES

1. Wang, M., Cao, R., Zhang, L., Yang, X., Liu, J., Xu, M., ... & Xiao, G. (2020). Remdesivir and chloroquine effectively inhibit the recently emerged novel coronavirus (2019-nCoV) in vitro. *Cell research*, *30*(3), 269-271.
2. Li, G., & De Clercq, E. (2020). Therapeutic options for the 2019 novel coronavirus (2019-nCoV). *Nat Rev Drug Discov*. 2020;19(3):149-150. doi:10.1038/d41573-020-00016-0
3. Narkhede, R. R., Cheke, R. S., Ambhore, J. P., & Shinde, S. D. (2020). The molecular docking study of potential drug candidates showing anti-COVID-19 activity by exploring of therapeutic targets of SARS-CoV-2. *EJMO*. 2020; 4(3): 185-195
4. Zumla, A., Chan, J. F., Azhar, E. I., Hui, D. S., & Yuen, K. Y. (2016). Coronaviruses—drug discovery and therapeutic options. *Nature reviews Drug discovery*, *15*(5), 327-347.
5. Dong, L., Hu, S., & Gao, J. (2020). Discovering drugs to treat coronavirus disease 2019 (COVID-19). *Drug discoveries & therapeutics*, *14*(1), 58-60.
6. McKee, D. L., Sternberg, A., Stange, U., Laufer, S., & Naujokat, C. (2020). Candidate drugs against SARS-CoV-2 and COVID-19. *Pharmacological Research*, 104859.
7. Gautret, P., Lagier, J. C., Parola, P., Meddeb, L., Mailhe, M., Doudier, B., ... & Honoré, S. (2020). Hydroxychloroquine and azithromycin as a treatment of COVID-19: results of an open-label non-randomized clinical trial. *International journal of antimicrobial agents*, 105949.

8. Liu, J., Cao, R., Xu, M., Wang, X., Zhang, H., Hu, H., ... & Wang, M. (2020). Hydroxychloroquine, a less toxic derivative of chloroquine, is effective in inhibiting SARS-CoV-2 infection in vitro. *Cell discovery*, *6*(1), 1-4.
9. De Oliveira, D. B., & Gaudio, A. C. (2000). BuildQSAR: a new computer program for QSAR analysis. *Quantitative Structure‐ Activity Relationships: An International Journal Devoted to Fundamental and Practical Aspects of Electroanalysis*, *19*(6), 599-601.
10. Hospital, A., Andrio, P., Fenollosa, C., Cicin-Sain, D., Orozco, M., & Gelpí, J. L. (2012). MDWeb and MDMoby: an integrated web-based platform for molecular dynamics simulations. *Bioinformatics*, *28*(9), 1278-1279.
