# Supplementary material for: Molecular docking study of potential phytochemicals and their effects on the complex of SARS-CoV2 spike protein and human ACE2
Source: Sci Rep. 2020 Oct 19;10:17699. doi: 10.1038/s41598-020-74715-4 (PMC7573581; doi:10.1038/s41598-020-74715-4)
Supplement: Supplementary file 3 — Supplementary Information 3 [file 41598_2020_74715_MOESM3_ESM.pdf]

Molecular docking study of potential phytochemicals and their effects on the complex of SARS-CoV2 spike protein and human ACE2

Anamika Basu[1], Anasua Sarkar[2,*], Ujjwal Maulik[3]

[1] *Department of Biochemistry, Gurudas College, India, Email: basuanamikaami@gmail.com*

[2] *Computer Science and Engineering Department, Jadavpur University, India, Email: anasua.sarkar@jadavpuruniversity.in*

[3] *Computer Science and Engineering Department, Jadavpur University, India, Email: umaulik@cse.jdvu.ac.in*

Supplementary file S3

The modulation of hesperidin of ACE2 protein prevents its interaction with spike protein. It has been proved by a simple in silico experiment. This in silico experiment is performed with this protocol:

1. Docking hesperidin and hydroxychloroquine with ACE-2 with their targeted binding at modulator site.

Results

Using Dockthor web server [48] molecular docking studies of hesperidin and hydroxychloroquine with ACE-2 is performed and results are shown in Table S3.1 and Figure S3.1.

Table S3.1 Binding affinity of ACE2 protein and different compounds as ligands

**Compound no.**	**Name of compounds**	**Affinity (Kcal/mole)**	**Total energy**	**VdW energy**	**Electrostatic energy**
1	Hesperidin	-9.167	55.969	-29.393	-22.905
2.	Hydroxychloroquine	-7.961	19.996	-11.079	-23.260

(a) (b)

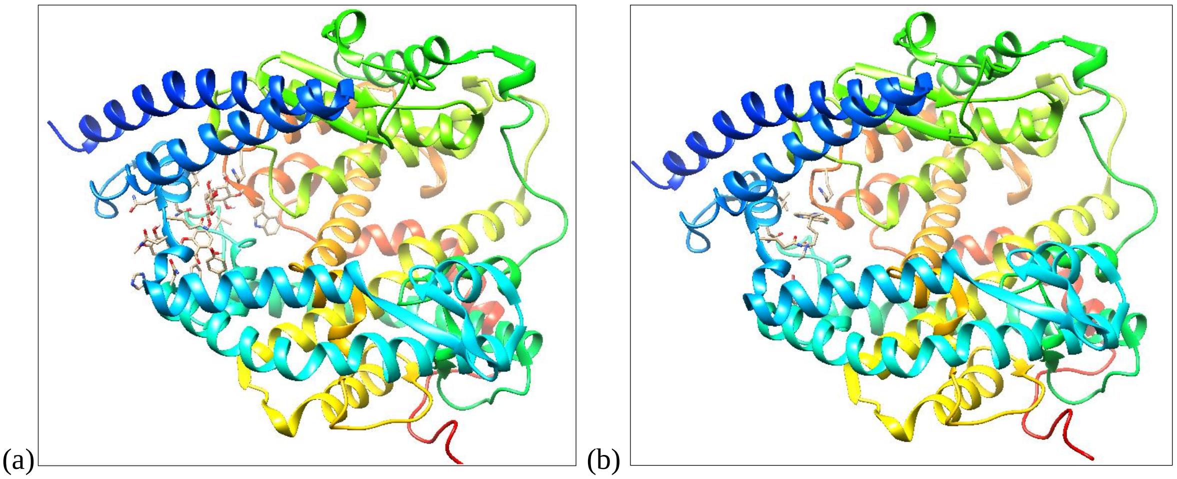

Figure S3.1(a) Docking structure of hesperidin with ACE-2 at the modulator site (b) Docking structure of hydroxychloroquine with ACE-2 at the modulator site

2. Docking of ACE2 and SARS CoV 2 spike protein separately.

Results

By using ClusPro [27] web server, docking structure of A chain of human ACE2 receptor, which binds with spike protein fragment, is obtained. SARS CoV2 spike protein binds with human ACE2 receptor protein with binding energy -779.8 Kcal/mole. [as mentioned in main manuscript]

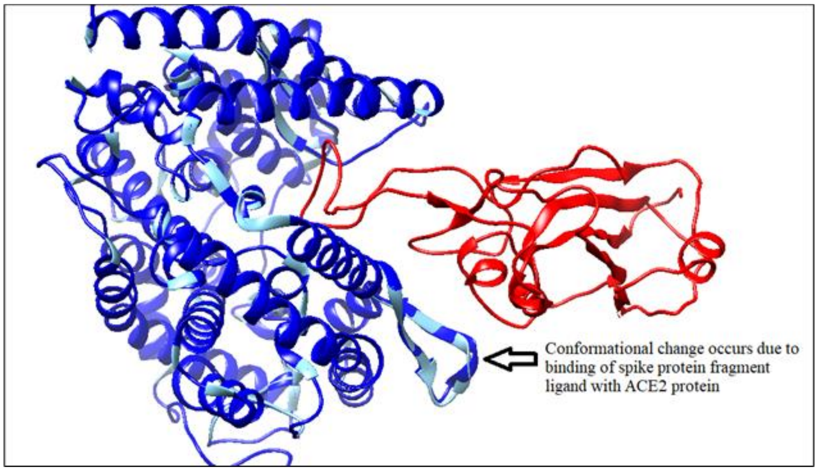

Figure 2 Docking structure of ACE2 and SARS CoV 2 spike protein fragment

3. Finally, Docking of Hesperidin- ACE2 protein complex and HCQ-ACE2 protein complex derived from first step with SARS CoV2 spike protein fragment.

Result:

(a) 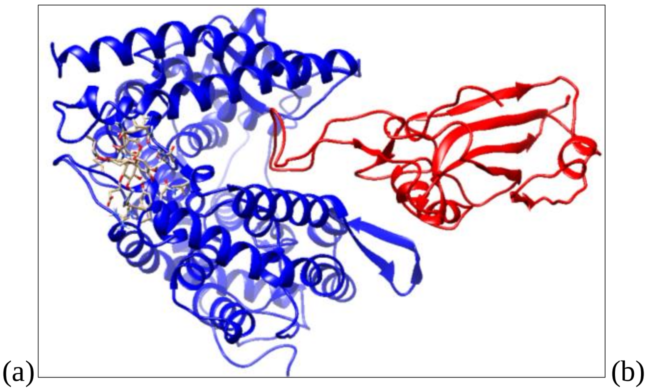 (b) 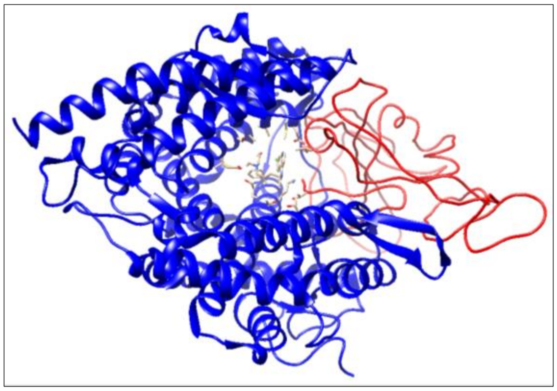

Figure S3.3 (a) Docking structure of Hesperidin- ACE2 protein complex with spike protein fragment (b) Docking structure of Hydroxychloroquine- ACE2 protein complex with spike protein fragment

Docking studies in step 3, shows that in presence of hesperidin, the binding energy of S protein fragment with ACE2 A chain changes from -779.8 Kcal/mole to -677.6 Kcal/mole. At the same time, the binding energy of hydroxychloroquine- ACE2 protein complex with spike protein fragment deviates from -779.8 Kcal/mole to -693.3 Kcal/mole. A significant decrease in binding potential in the third step compared to second step, is observed. The modulation of Hesperidin of ACE2 protein should now prevent its interaction with spike protein.

In both cases the values of the binding energy of SARS CoV2 spike protein fragment with human ACE2 receptor protein, decrease in presence of two modulators i.e. hesperidin and hydroxychloroquine. Due to presence of natural product hesperidin, the bound structure of S protein fragment with ACE2 A chain, becomes more unstable, compared to that of hydroxychloroquine. So, it can be concluded that the phytochemical hesperidin is more efficient as antiviral agent compared to hydroxychloroquine.
